# The microbiota extends the reproductive lifespan of mice by safeguarding the ovarian reserve

**DOI:** 10.1016/j.chom.2025.09.006

**Published:** 2025-09-25

**Authors:** Sarah K. Munyoki, Julie P. Goff, Amanda Reshke, Erin Wilderoter, Nyasha Mafarachisi, Antonija Kolobaric, Yi Sheng, Steven J. Mullett, Gabrielle E. King, Jacob D. DeSchepper, Richard J. Bookser, Carlos A. Castro, Stacy L. Gelhaus, Mayara Grizotte-Lake, Kathleen E. Morrison, Anthony J. Zeleznik, Timothy W. Hand, Miguel A. Brieño-Enriquez, Eldin Jašarević

**Affiliations:** 1 Department of Obstetrics, Gynecology, and Reproductive Sciences, University of Pittsburgh School of Medicine, Pittsburgh, PA, USA; 2 Department of Computational and Systems Biology, University of Pittsburgh School of Medicine, Pittsburgh, PA, USA; 3 Magee-Womens Research Institute, Pittsburgh, PA, USA; 4 Department of Pharmacology & Chemical Biology, University of Pittsburgh School of Medicine, Pittsburgh, PA, USA; 5 Health Sciences Mass Spectrometry Core, University of Pittsburgh School of Medicine, Pittsburgh, PA, USA; 6 Taconic Biosciences, Resanssler, NY, USA; 7 Department of Psychology, West Virginia University, Morgantown, WV, USA; 8 University of Pittsburgh Gnotobiotic Animal Core, Office of Research, Health Sciences, University of Pittsburgh School of Medicine, Pittsburgh, PA, USA; 9 Department of Pediatrics, UPMC Children’s Hospital of Pittsburgh, University of Pittsburgh, Pittsburgh, PA, USA; 10 Lead contact

## Abstract

Infertility affects one in six people, but the underlying mechanisms remain unclear. We show that the microbiota governs female reproductive longevity in mice. Germ-free mice have fewer primordial follicles, increased atresia, and ovarian fibrosis, leading to smaller litters, fewer offspring, and a shorter reproductive lifespan. Germ-free mice are born with a similar ovarian reserve but display excessive activation, impaired progression, and increased atresia during post-natal development. Microbiome colonization during a critical post-natal window rescues premature ovarian reserve loss by normalizing follicle kinetics and gene expression patterns. These changes parallel increased short-chain fatty acids (SCFAs), and SCFA administration mitigates ovarian dysfunction in germ-free mice. Similar oocyte dysfunction occurred in conventionally raised mice fed a high-fat diet, but additional dietary fiber helped preserve oocyte quality and embryo competence. Thus, host-microbe interactions shape female fertility, and microbiota-targeted interventions may offer strategies to address reproductive disorders.

## In brief

Munyoki et al. demonstrate that gut microbiota extend the female reproductive lifespan by safeguarding the ovarian reserve. Germ-free mice undergo accelerated primordial follicle loss and reduced reproductive capacity that are restored upon post-natal colonization. Microbial SCFAs contribute to protection, and dietary fiber supplementation maintains oocyte quality under obesogenic diets.

## Graphical abstract

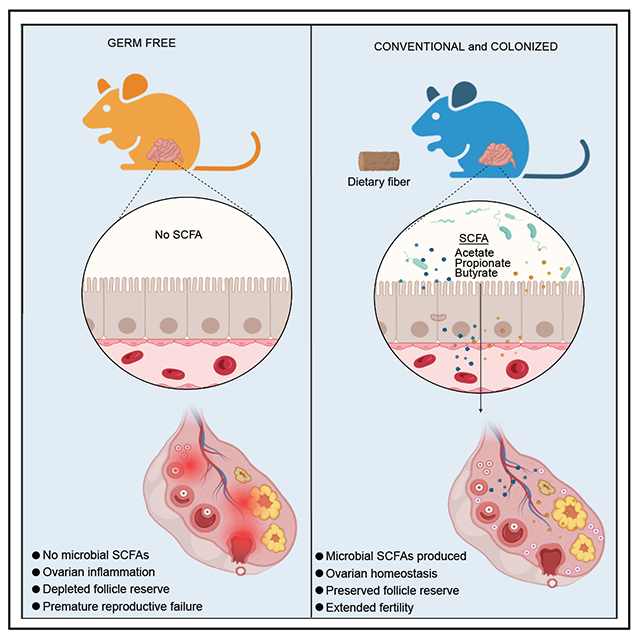

## INTRODUCTION

Infertility affects one in six people and continues to increase worldwide.^1^ Similar alarming trends are reported across the animal kingdom,^2^ underscoring an urgent need to identify unifying principles and mechanisms governing reproductive health. Despite advances in assisted reproductive technologies, fundamental regulators of fertility remain unclear. The microbiota orchestrates host metabolism, immunity, and development,^3–6^ and emerging evidence links microbial communities to reproduction and pregnancy outcomes.^7–15^

Across the animal kingdom, microbes mediate trade-offs between growth and reproduction.^16^ In invertebrates, specific bacteria are required for sexual maturation. Mosquitoes, for example, fail to pupate without certain bacteria but resume development upon microbial colonization.^17^ Similarly, oogenesis is disrupted in axenic nematodes, honeybees, fruit flies, and wasps.^18–22^ This process can be restored through bacterial colonization, with the microbiota providing the substrates necessary for egg formation.^20^

The relationship between the microbiota and reproductive capacity has been a long-standing interest in mammals. Poor reproductive outcomes in germ-free (GF) mice^9,10,23^ demonstrate that microbiota influences mammalian reproduction.^24^ Antibiotic-mediated disruption of the microbiota impairs male fertility,^25^ but the role of the microbiota in female fertility remains underexplored. This knowledge gap is especially concerning given that metabolic disorders like obesity, which change the microbiota, are associated with higher rates of reproductive issues and lower quality of life in women.^26–28^ Environmental factors that disrupt the microbiota, such as antibiotics, diet, stress, and exposure to pollutants, are also linked to reproductive dysfunction,^29–31^ yet underlying mechanisms remain unknown. Understanding how microbial signals influence ovarian biology could open new treatment options in a field with few novel therapies.

Here, we test the hypothesis that microbiota-derived signals regulate female reproductive function. Our study addresses a fundamental and long-standing question in reproductive biology regarding the preservation of the ovarian reserve^32,33^—a finite and non-renewable pool of oocytes—and provides new insights into the microbial regulation of female reproductive health.

## RESULTS

### The microbiota is required for extending the reproductive lifespan in mice

**Figure 1.**
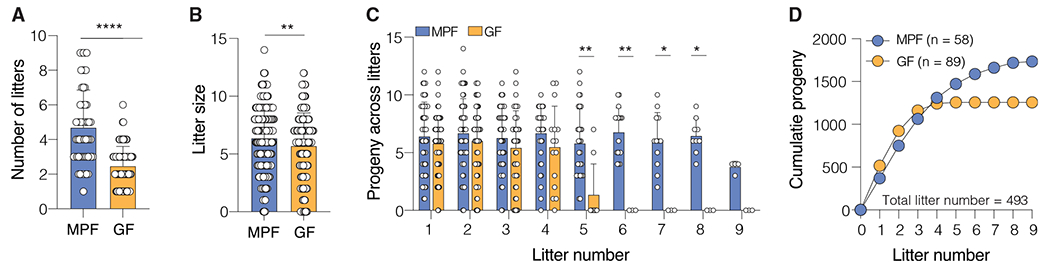
The microbiota extends the reproductive lifespan in mice (A) Lifetime litters in MPF (*n* = 58) and GF (*n* = 89) mice. t test, ****p < 0.0001. (B) Mean litter size (273 MPF litters; 220 GF litters). t test, **p < 0.01. (C) Progeny per sequential litter. Sample sizes per litter: 1 (MPF *n* = 58, GF *n* = 89), 2 (MPF *n* = 57, GF *n* = 68), 3 (MPF *n* = 50, GF *n* = 44), 4 (MPF *n* = 37, GF *n* = 15), 5 (MPF *n* = 28, GF *n* = 9), 6 (MPF *n* = 17, GF *n* = 0), 7 (MPF *n* = 12, GF *n* = 0), 8 (MPF *n* = 9, GF *n* = 0), 9 (MPF *n* = 4, GF *n* = 0). Mixed-effects ANOVA with Šidák’s test, *p < 0.05, **p < 0.01. (D) Cumulative progeny across 9 litters (total litters = 493). All breeding data from C57BL/6N Tac mice were obtained using standardized breeding protocols. Mating pairs were initiated at 8 weeks and continued until 9 litters were produced or until 32 weeks. Males remained housed with females. Data are mean ± SD. Average litter interval: 21–28 days; gestation: 19–21 days. MPF, murine pathogen-free; GF, germ-free. See also Figure S1.

In the 1970s, researchers discovered that GF mice had smaller litters compared with those of conventional mice.^34,35^ Building on these findings, we analyzed lifetime breeding records from 493 litters of conventionally raised, murine pathogen-free (MPF) and GF C57BL/6N Tac mice. MPF mice averaged 4.7 litters, while GF mice averaged 2.5 litters (Figure 1A), with fewer pups per litter in GF mice (Figure 1B). MPF mice produced offspring through nine litters, while GF mice showed declining fertility by litter five and infertility by litter six (Figures 1C and 1D). Undersampling with 1,000 permutations confirmed the robustness of the reproductive lifespan phenotype (Figure S1A).

**Figure 2.**
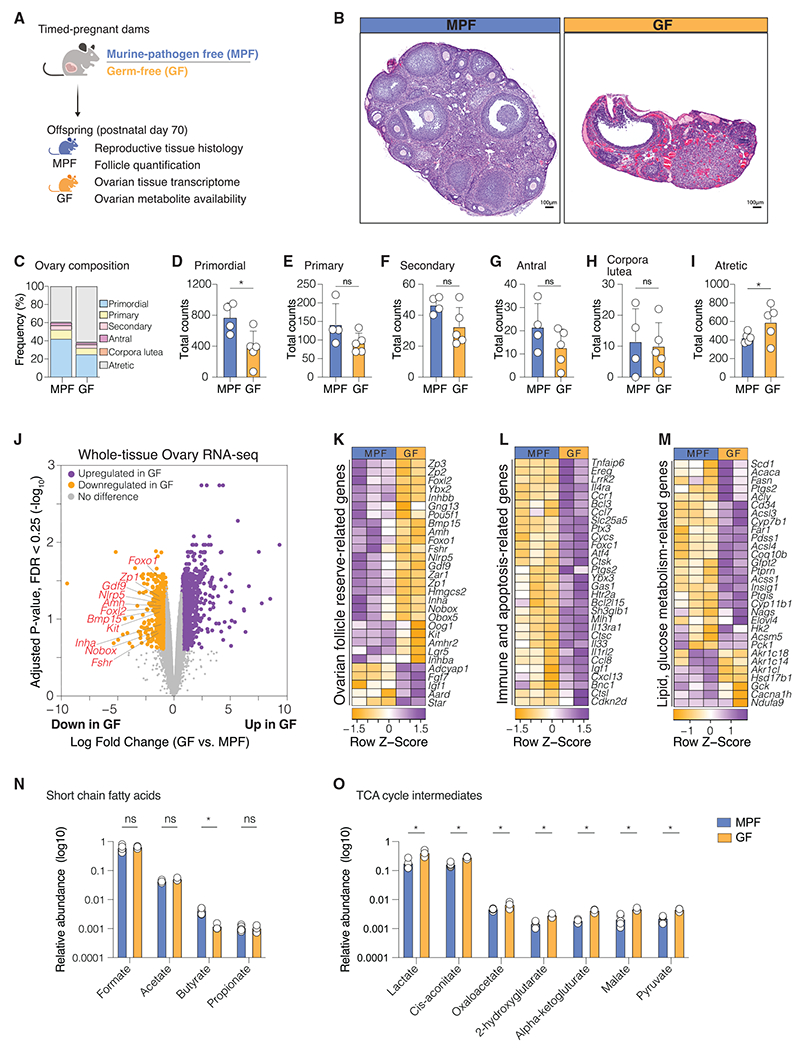
The gut microbiota impacts reproductive capacity through the regulation of ovarian follicle dynamics (A) Experimental design: tissues from 10-week-old (P70) MPF and GF offspring were collected for histology (H&E), follicle counts, whole-tissue RNA-seq, and targeted ovarian metabolomics. on-littermate females were used for analyses. Schematic in BioRender. (B) Representative ovarian histology (H&E). Scale bars: 100 μM. (C) Ovarian follicle composition in MPF (*n* = 4) and GF (*n* = 5) mice. (D–I) Counts of primordial (D), primary (E), secondary (F), antral (G), corpora lutea (H), and atretic (I) follicles in MPF (*n* = 4) and GF (*n* = 5) mice. t test, *p < 0.05; ns, not significant. (J) Volcano plot of differentially expressed ovarian genes, with key ovarian reserve genes annotated. FDR < 0.25 (*n* = 2 GF, 3 MPF). (K–M) Heatmaps of genes related to (K) ovarian reserve maintenance, (L) immunity, and (M) metabolism (*n* = 2 GF, 3 MPF). (N) SCFAs in ovaries from MPF (*n* = 4) and GF (*n* = 5) mice. t test per metabolite, *p < 0.05; ns, not significant. (O) TCA cycle intermediates in ovaries from MPF (*n* = 4) and GF (*n* = 5) mice. t test per metabolite, *p < 0.05. Data are mean ± SD. MPF, murine pathogen-free; GF, germ-free. See also Figure S1.

To identify the biological basis for the reduced GF reproductive capacity, we examined reproductive tissues from 10-week-old adult (post-natal day 70 [P70]) MPF and GF mice (Figure 2A). Histopathological analysis of testes from MPF and GF males revealed intact spermatogenesis, including the presence of Sertoli cells, spermatogonia, spermatocytes, and spermatids in the lumen of seminiferous tubules (Figure S1B), replicating previous work in GF males.^34,36^ Additionally, GF males retain the ability to fertilize eggs and sire offspring at similar rates to conventional controls, despite reported abnormalities in the epididymis.^37^

In contrast, GF ovaries exhibited hemorrhagic lesions, collagen accumulation, and fibrosis (Figures 2B, S1C, and S1D). Quantification of ovarian follicles revealed a specific impact on the primordial follicle pool, referred to as the ovarian reserve^38,39^ (Figures 2C and 2D). Primordial follicles in GF ovaries were reduced by 50%, compared with MPF females (MPF: 766 vs. GF: 376) (Figure 2D). Counts of primary, secondary, and antral follicles were similar between groups (Figures 2E–2G), suggesting that the gonadotropin-dependent phase of follicle development is intact in GF mice.^40,41^ The number of corpora lutea was also comparable, indicating preserved ovulation (Figure 2H). GF ovaries also had increased atretic follicles, compared with MPF females (Figure 2I).

To determine molecular changes linked to ovarian reserve loss in GF females, we conducted whole-tissue RNA sequencing (RNA-seq) on P70 MPF and GF ovaries. This analysis revealed over 600 differentially expressed genes (DEGs) (false discovery rate [FDR] < 0.25), including downregulation of genes involved in maintaining quiescence, initiating activation, and promoting the survival of primordial follicles (*Nobox*, *Kit*, *Amh*, *Foxl2*, *Gdf9*, *Fshr*, *Figla*)^42–46^ (Figure 2J). Gene Ontology (GO) analysis identified enrichment of pathways involved in ovarian reserve, immunity, metabolism, and tissue remodeling (Figures 2K–2M and S3A). To link transcriptional changes with tissue pathology, we performed Masson’s trichrome staining and observed extensive collagen fiber deposition in GF ovaries, which was absent in MPF ovaries^47^ (Figures S1C and S1D). The transcriptional changes were also reflected in metabolic shifts in ovarian tissue, including reduced levels of the short-chain fatty acid (SCFA) butyrate and an increased abundance of measured tricarboxylic acid (TCA) cycle intermediates in GF, compared with MPF mice (Figures 2N and 2O).

Since the brain controls key aspects of ovarian function, we measured *Kiss1* and *Pdyn* expression in the arcuate nucleus of the hypothalamus in P70 GF and MPF females. These genes are co-expressed in neurons that regulate fertility and pregnancy.^48–51^ Using quantitative reverse transcription PCR (RT-qPCR), we found no differences in *Kiss1* or *Pdyn* mRNA transcript levels between GF and MPF females (Figures S1A and S4B). This result, along with our characterization of ovaries, indicates that the reproductive deficits in GF female mice likely stem from effects on ovarian tissue. However, since the hypothalamic-pituitary-ovarian axis is a circuit that is dynamically regulated across the lifespan,^52^ we cannot rule out hypothalamic involvement beyond the endpoints measured.

### The presence of microbiota prevents premature loss of the ovarian reserve during the post-natal period

**Figure 3.**
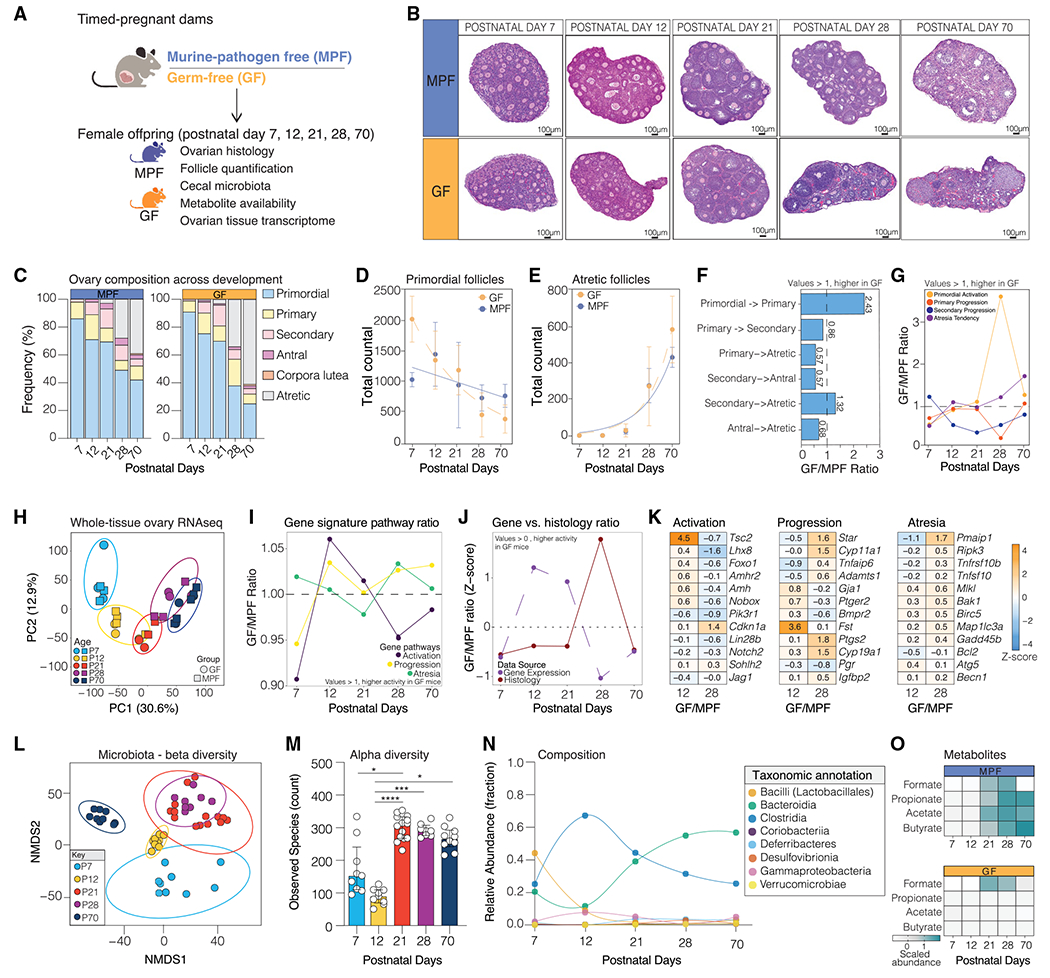
Post-natal microbiota maturation shapes ovarian follicle development (A) Experimental design: female offspring were collected at P7, P12, P21, P28, and P70 for analysis of ovarian histology, follicle counting, cecal microbiota profiling, and metabolomics. One female offspring per litter was used at each time point. Schematic created in BioRender. (B) Representative ovarian histology across ages (H&E). Scale bars: 100 μM. (C) Ovarian follicle composition across ages. Sample sizes: P7 (MPF *n* = 3, GF *n* = 4), P12 (MPF *n* = 4, GF *n* = 4), P21 (MPF *n* = 4, GF *n* = 4), P28 (MPF *n* = 4, GF *n* = 4), P70 (MPF *n* = 4, GF *n* = 5). (D) Primordial follicle kinetics across ages. MPF mice show linear decline (y = 1,362.9 – 126.2×, R^2^ = 0.47), while GF mice exhibit exponential decay (y = 3,054.5e^(−0.394x)^, R^2^ = 0.95). (E) Atretic follicle counts across ages with exponential model fits (GF: R^2^ = 0.97, MPF: R^2^ = 0.92). Sample size as in (C). (F) Compartmental modeling shows higher primordial activation with reduced progression in GF mice. Sample size as in (C). (G) Efficiency ratios (primary/primordial; secondary/primary; antral/secondary; atretic/total); values > 1 indicate higher GF ratios. Sample size as in (C). (H) Principal-component analysis showing clustering by age (PC1, 30.6%) and microbiota status (PC2, 12.9%). Sample sizes: P7 (MPF *n* = 3, GF *n* = 3), P12 (MPF *n* = 3, GF *n* = 4), P21 (MPF *n* = 4, GF *n* = 4), P28 (MPF *n* = 3, GF *n* = 3), P70 (MPF *n* = 3, GF *n* = 5). (I) Pathway signatures reveal activation-progression imbalance across development. Sample size as in (H). (J) Comparison of gene expression vs. histological ratios (*Z* scores) shows a disconnect between molecular and morphological changes. Sample size as in (C) for histology and (H) for gene expression. (K) Heatmap of follicle activation, progression, and atresia genes between P12 and P28, shown as GF/MPF fold change. Sample size as in (H). (L) Non-metric multidimensional scaling analysis of microbiota across ages in MPF mice. PERMANOVA, *p* < 0.001 for all comparisons. Sample sizes: P7 (MPF *n* = 9), P12 (MPF *n* = 9), P21 (MPF *n* = 14), P28 (MPF *n* = 8), P70 (MPF *n* = 9). (M) Alpha diversity across ages in MPF mice. Kruskal-Wallis test for non-parametric data followed by Dunn’s test, *p < 0.05, ***p < 0.001, ****p < 0.0001. Sample size as in (L). (N) Fractional abundance of major taxa across ages. Sample size as in (L). (O) Cecal SCFA abundance across ages. Sample size as in (L). Data are mean ± SD, unless otherwise noted. MPF, murine pathogen-free; GF, germ-free; SCFA, short-chain fatty acid. See also Figures S2 and S3.

To determine the timing of when microbiota impacts ovarian dynamics, we quantified ovarian follicles at various post-natal (P) stages (7, 12, 21, 28, 70)^38,39,53–56^ (Figure 3A). These time points were selected to capture key developmental stages: early primordial follicle formation (P7),^57,58^ pre-pubertal development (P12),^59^ pubertal transition (P21),^59^ post-pubertal maturation (P28),^59^ and adult reproductive function (P70).^59^ Histology showed no differences in MPF and GF ovarian morphology during early development, from P7 to P12. Signs of pathology, such as hemorrhagic lesions, fibrosis, and decreased ovarian volume, appeared at P28 and persisted at P70 (Figure 3B). Follicle counts revealed that GF mice had twice as many primordial follicles at P7 (MPF: ~1,000 vs. GF: ~2,000). This early difference disappeared by P12 when primordial follicle counts were similar between groups (MPF: ~1,500 vs. GF: ~1,350) and remained comparable at P21 (MPF: ~950 vs. GF: ~1,100). By P28, GF primordial follicle numbers started to decline (MPF: ~700 vs. GF: ~450) and continued through P70, where significant differences appeared (MPF: ~750 vs. GF: ~350) (Figures 3C and S2A–S2F).

We then modeled ovarian follicle dynamics using linear, exponential, and power functions, comparing fits with the Akaike information criterion (AIC).^60–63^ This approach identified unique follicle loss patterns between groups (Table S1). For MPF mice, primordial follicle loss followed linear kinetics (R^2^ = 0.47, AIC = 72.6) with a decline of 126.2 follicles per time point. GF mice exhibited exponential decay of primordial follicles (R^2^ = 0.95, AIC = 69.8), indicating accelerating loss over time (Figure 3D). Atretic follicle accumulation followed exponential kinetics in both MPF (R^2^ = 0.92, AIC = 59.3) and GF (R^2^ = 0.97, AIC = 56.2) mice, with GF ovaries showing more rapid accumulation by P70 (Figure 3E). Together, these models demonstrate that the absence of microbiota reshapes post-natal follicle dynamics.

To determine why GF mice lost more primordial follicles, we examined transition dynamics using two complementary approaches: (1) a compartmental model analysis of stage-specific transition rates and (2) direct comparisons of follicle stage ratios across post-natal ages.^64^ The model assumes unidirectional progression through discrete stages with first-order kinetics, relatively constant transition rates, and equal probabilities of advancement or atresia within each stage. The analysis revealed microbiota-dependent differences (Figures 3F and S2G). GF mice exhibited a 2.4-fold higher rate of primordial follicle activation (0.42 vs. 0.17 per day) compared with MPF mice, but this acceleration was not compensated at later stages. Instead, GF mice showed reduced progression from primary to secondary follicles (GF/MPF ratio = 0.86) and decreased transition from secondary to antral stages (GF/MPF ratio = 0.57). This developmental bottleneck was linked to higher rates of atresia, with GF mice exhibiting increased atresia rates for secondary follicles (GF/MPF ratio = 1.32).

We then compared direct ratios between adjacent follicle stages across post-natal time points (Figures 3G and S2H–S2K). These measurements confirmed our model results, showing that the activation ratio (primary/primordial counts) in GF compared with MPF mice increased starting at P12 and peaked at P28. At the same time, the primary progression ratio (secondary/primary counts) decreased by P28, and the secondary progression ratio (antral/secondary counts) showed similar decreases. The atresia tendency (atretic/total follicles) increased in GF mice, showing higher ratios compared with MPF mice. By adulthood (P70), these efficiency ratios began to normalize, but by then, the primordial follicle pool had already been depleted in GF mice due to earlier developmental events. These results indicate an imbalance between follicle activation and growth in GF mice, characterized by excessive activation, impaired progression, and increased atresia.

Having established that the absence of the microbiota disrupts ovarian follicle dynamics across development, we next examined underlying molecular signatures. We profiled wholeovary RNA-seq across matching post-natal ages and related these data to our histological findings. Principal-component analysis showed a clear separation of samples by age along PC1 (30.6% of variation) (Figure 3H). Differential expression analysis (FDR < 0.25) revealed a progressive accumulation of genes changes between GF and MPF ovaries, starting with a few differences at P7 (~200 genes) and reaching peak differences at P28 (~1,300 genes), coinciding with the timing of our observed histological changes (Figure S2L). GO analysis of downregulated genes in GF compared with MPF mice identified pathways involved in cell fate commitment, oocyte maturation, energy balance, and immune signaling (Figure S7M), whereas upregulated genes in GF ovaries did not reveal enrichment for specific pathways. These results indicate that the lack of microbiota leads to progressive transcriptional dysregulation that precedes follicle depletion.

To define gene networks affected by the absence of the microbiota, we focused on pathways governing follicle activation (including 35 genes such as *Foxo3*, *Pten*, *Nobox*, *Kit/Kitl*, and *Amh/Amhr2*), progression (including 50 genes such as *Fshr*, *Gdf9*, *Bmp15*, *Star*, *Esr1*, *Igf1*, and *Igfr1*), and atresia (including 46 genes such as *Bax/Bcl2* family members, caspases, and autophagy regulators) (see Table S2 for gene list and references). For each pathway, we generated expression signatures by averaging normalized expression values of pathway genes across all ovarian samples. Activation-related gene expression in GF ovaries increased from P7, peaked at P12, and declined by P28, while progression and atresia-related signatures fluctuated across post-natal time points (Figure 3I). To integrate molecular and histological findings, we compared activation-to-progression ratios from both datasets (Figure 3J). Standardized comparisons (*Z* scores) of these ratios revealed a temporal disconnect between gene expression and morphological changes as captured by histological quantification (Figure 3J). We found that gene expression imbalance was most pronounced at P12, preceding the morphological imbalance detected histologically at P28. These results show that molecular alterations emerge before histological evidence of disrupted follicle dynamics.

To probe the molecular basis of the activation-progression imbalance, we analyzed genes with the strongest differential expression between GF and MPF mice at P12–P28 (Figure 3K). In the activation pathway, *Tsc2*, a negative regulator of mammalian target of rapamycin (mTOR) signaling, showed higher differential expression in GF mice at P12 (4.5-fold GF/MPF) but downregulation at P28 (−0.7-fold GF/MPF). Similar inversions were observed for dormancy-associated genes, including *Amh*, *Amhr2*, *Foxo1*, and *Lhx8*. In the progression pathway, steroidogenic regulators (*Star*, *Cyp11a1*) shifted from lower expression at P12 to higher expression at P28, while genes mediating cell-cell communication (*Gja1*, *Ptger2*) and growth factor signaling (*Bmpr2*) showed the opposite trend. Consistent with increased atresia, the pro-apoptotic factor *Pmaip1* shifted from reduced (−1.1-fold GF/MPF) to elevated expression (1.7-fold GF/MPF) between P12 and P28, while expression of the anti-apoptotic *Bcl2* remained low in GF ovaries. These findings show that microbiota-dependent signals coordinate complex temporal regulation of activation, progression, and survival pathways.

### Post-natal gut microbiota maturation aligns with critical periods of ovarian follicle regulation

To track gut microbiota dynamics alongside ovarian development, we profiled the cecal contents of MPF female mice across various post-natal ages P7–P70 using 16S rRNA marker gene sequencing. Non-metric multidimensional scaling analysis revealed age-dependent shifts in community structure (Figure 3L). Statistical comparisons confirmed differences in community structure (PERMANOVA, *p* < 0.001 for all comparisons), with the most distinct shifts occurring from breastfeeding to the earliest stages of the weaning transition (P7–P12, *R*^2^ = 0.25) and transition to adulthood (P28–P70, *R*^2^ = 0.39), while juvenile time points (P21–P28) showed more modest differences (*R*^2^ = 0.12) (Figure 3L). Alpha diversity (observed amplicon sequence variant [ASV] counts, rarefied to a minimum depth of 4,511 reads) increased significantly from P21 onward (Figure 3M), marking the establishment of a more complex, adult-like microbiota. These findings demonstrate that major shifts in gut community composition coincide with the window when ovarian follicle dynamics are altered in GF mice.

To identify the taxa that shift during post-natal development, we performed differential abundance analysis (ALDEx2)^65^ from P7 to P70 (Figure 3N; Table S3). At P7, cecal communities were dominated by breast milk-associated genera including *Lactobacillus*, *Streptococcus*, and *Enterococcus*, consistent with prior reports.^7,8,15,66–71^ The weaning transition began at P12, marked by the emergence of *Clostridia* members, such as *Lachnospiraceae* ^66^ (Figure 3N; Table S3). This transition coincided with the onset of solid food intake, which introduces dietary polysaccharides and complex carbohydrates into the intestine, supporting bacterial fermentation and production of microbial metabolites like SCFAs.^72^

These microbial shifts coincide with coordinated physiological and behavioral changes, including feeding behavior, digestive capabilities, metabolism, and neurological development.^72^ Between P12 and P21, pups open their eyes and ears, start exploring their environment, and initiate coprophagy driven by maternal pheromones.^72–75^ Maternal fecal particles are readily recovered from pup stomachs before food particles, suggesting exploratory behavior promotes maternal microbiota transfer.^73,74,76^ As expected, weaning was marked by microbial diversification, with the expansion of *Bacteroidia* and *Clostridia*, including *Bacteroides*, *Muribaculaceae*, *Lachnospiraceae*, and *Clostridiaceae*, as well as segmented filamentous bacteria (*Candidatus Arthromitus*) (Table S3). At the same time, breast milk-adapted genera *Ligilactobacillus*, *Enterococcus*, and *Streptococcus* declined. By P21, transition to solid food aligned with an increase in microbial richness. Shifts from P21 to P28 were modest, including the emergence of *Desulfovibrio*, while maturation from P28 to P70 involved further diversification of *Lachnospiraceae* and *Bacteroidia*, with *Muribaculaceae* becoming the dominant taxa in adult MPF female mice (Table S3).

To gain species-level resolution and assess functional potential, we performed shotgun metagenomic sequencing of cecal samples. This confirmed post-natal expansion of SCFA producers, including *Bacteroidia* (*Bacteroides caccae*, *Butyricimonas faecalis*, and *Muribaculum* species), *Clostridia* (including known butyrate-producers *Anaerostipes hallii*, *Clostridium butyricum*), and *Erysipelotrichia* (*Faecalibaculum rodentium* and *Roseburia hominis*) (Figure S2N). Gene-level analysis showed enrichment of microbial metabolic pathways involved in SCFA production, starting at P12 and persisting into adulthood (Figure S2O). Comparing physiological parameters across post-natal time points revealed significant differences in body mass and cecal mass between MPF and GF mice during post-natal development (Figures S3A and S3B). GF mice had larger ceca and higher cecal mass-to-body mass ratios (Figure S3C). These differences explained the body mass variation, as body mass after cecal removal was similar between groups, consistent with recent findings^77^ (Figure S3D). Targeted metabolomics confirmed that MPF mice accumulated cecal SCFAs from P12 onward, whereas GF mice did not (Figures 3O and S3E–S3L). These findings show that the post-natal expansion of SCFA-producing obligate anaerobes during the weaning period (P12–P28) coincides with the development period when GF mice exhibit an imbalance between primordial follicle activation and progression in GF mice.

### Microbiota colonization is sufficient to prevent premature loss of the ovarian reserve

**Figure 4.**
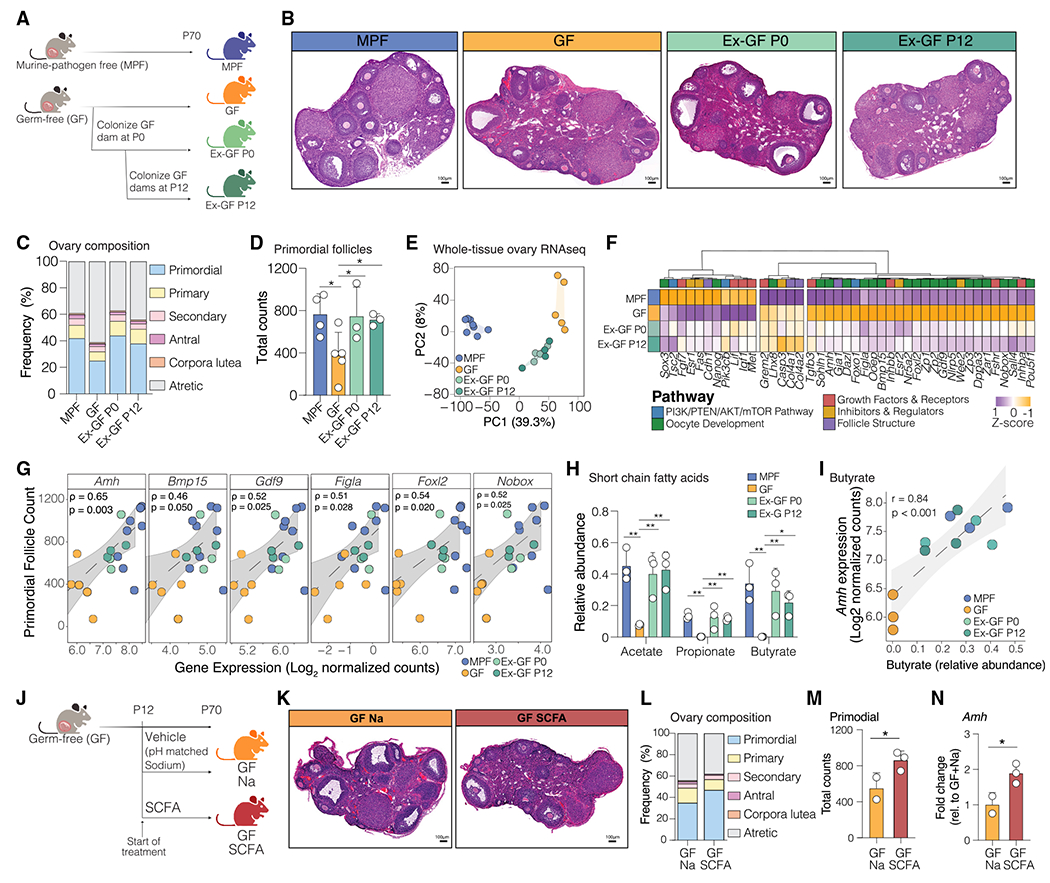
Post-natal microbiota colonization during the weaning transition prevents premature ovarian reserve loss (A) Experimental design: GF dams were colonized with intestinal microbial contents from adult P70 MPF donors at birth (Ex-GF P0) or at weaning transition onset (Ex-GF P12). Colonized offspring were analyzed at P70 alongside MPF and GF mice. Schematic created in BioRender. (B) Representative ovarian histology for MPF, GF, Ex-GF P0, and Ex-GF P12 mice (H&E staining). Scale bars: 100 μM. (C) Ovarian follicle composition in MPF (*n* = 4), GF (*n* = 5), Ex-GF P0 (*n* = 3), and Ex-GF P12 (*n* = 3) mice. (D) Primordial follicle counts across groups. One-way ANOVA with Fisher’s least significant difference (LSD) test (*p < 0.05). Sample size as in (C). (E) Principal-component analysis of whole-tissue ovary RNA-seq data showing clustering by microbiota status (PC1, 39.3% variance). MPF (*n* = 8), GF (*n* = 5), Ex-GF P0 (*n* = 4), and Ex-GF P12 (*n* = 4) mice. (F) Heatmap showing clusters with different responses to colonization, grouped by pathway, and colored by *Z* score expression values. Sample sizes as in (E). (G) Correlations between ovarian gene expression and primordial follicle counts. Pearson correlation coefficients (r) and *p* values are shown for each gene. Sample sizes as in (E). (H) Cecal SCFA abundance across groups. One-way ANOVA with Tukey’s test (**p < 0.01). MPF (*n* = 3), GF (*n* = 3), Ex-GF P0 (*n* = 3), Ex-GF P12 (*n* = 3). (I) Correlation between ovarian *Amh* expression and cecal butyrate abundance (Pearson correlation, r = 0.84, *p* < 0.001). MPF (*n* = 3), GF (*n* = 3), Ex-GF P0 (*n* = 3), Ex-GF P12 (*n* = 3). (J) Experimental design: GF mice received SCFA mix (67.5 mM acetate, 40 mM butyrate, 25.9 mM propionate) or pH- and sodium-matched solution in drinking water from P12 to P70, and endpoints were assessed. (K) Representative ovarian histology for GF+Na and GF+SCFA mice (H&E). Scale bars: 100 μM. (L) Ovarian follicle composition in GF+Na (*n* = 2) and GF+SCFA (*n* = 3) mice. (M) Primordial follicle counts in GF+Na and GF+SCFA mice. Sample sizes as in (L). t test, *p < 0.05. (N) Ovarian *Amh* expression (RT-qPCR) in GF+Na and GF+SCFA mice. Sample sizes as in (L). t test, *p < 0.05. Data are mean ± SD. MPF, murine pathogen-free; GF, germ-free; Ex-GF, conventionalized GF mice; SCFA, short-chain fatty acid; Na, sodium. See also Figures S4 and S5.

We then assessed whether introducing microbiota at defined post-natal time points could rescue the GF ovarian phenotype. To enable vertical transmission, we colonized GF dams with intestinal contents from adult MPF donors at birth (P0) or at the onset of the weaning transition (P12) (Figure 4A). These time points were chosen to test whether colonization as late as P12 is sufficient for rescue of the ovarian phenotype. Engraftment was confirmed by 16S rRNA profiling and targeted metabolomics, which showed recovery of microbial diversity, community composition, and SCFA levels comparable to those in MPF dams (Figures S4A–S4C).

Histological analysis of P70 ovaries showed that either P0 and P12 colonization restored the primordial follicle pool to levels seen in MPF mice and reversed the morphological phenotypes observed in GF mice (Figures 4B–4D). This effect was specific to the primordial follicle pool, as numbers of growing follicles and corpora lutea were unchanged across groups (Figures S4D–S4H). Colonization at both time points prevented the collagen deposition and fibrosis characteristics in GF mice (Figure S4I). These findings demonstrate that introducing microbiota as late as P12 is sufficient to prevent premature depletion of the ovarian reserve, coinciding with the normal appearance of obligatory anaerobes during normal post-natal development (Figures 3F and 3G).

To define the molecular basis of rescue, we performed wholeovary RNA-seq at P70 across all groups. Principal-component analysis revealed distinct clustering, with microbiota status driving major variation (PC1, 39.3%) (Figure 4E). Differential expression analysis identified three distinct responses to colonization (Figure 4F). The first cluster normalized MPF-like expression patterns in both colonized groups, including key oocyte-specific transcription factors (*Pou5f1*, *Nobox*, *Foxl2*, *Sohlh1*), zona pellucida components (*Zp1*, *Zp2*, *Zp3*), oocyte-specific genes (*Zar1*, *Dppa3*, *Nlrp5*), and follicle regulators (*Gdf9*, *Bmp15*, *Amh*, *Figla*). The second cluster remained upregulated in GF and colonized mice, including growth factor signaling genes (*Met*, *Igf1*, *Lif*, *Pik3cb*), pluripotency factors (*Nanog*, *Sox3*), and hormone signaling mediators (*Esr1*). The third cluster remained downregulated in GF and colonized mice, including basement membrane components (*Col4a1*, *Col4a2*), apoptosis regulators (*Casp3*), and the oocyte transcription factor *Lhx8*.

To link transcriptional changes with ovarian phenotypes, we compared gene expression with follicle counts. Several regulatory genes showed strong positive correlations with primordial follicle counts, including *Amh* (r = 0.65, *p* = 0.003), *Bmp15* (r = 0.46, *p* = 0.050), *Gdf9* (*r* = 0.52, *p* = 0.025), *Figla* (*r* = 0.51, *p* = 0.028), *Foxl2* (*r* = 0.54, *p* = 0.020), and *Nobox* (*r* = 0.52, *p* = 0.025)^42,43,45,46,78–80^ (Figure 4G). These correlations indicate that post-natal microbiota colonization restores gene networks essential for ovarian reserve maintenance.

Beyond ovarian effects, colonization at P0 or P12 normalized the cecum-to-body mass ratio, gut microbiota, and SCFA levels, aligning them with those of MPF females (Figures 4H and S5A–S5H). Ovarian *Amh* expression correlated with cecal SCFA levels, including butyrate (*r* = 0.84, *p* < 0.001), acetate (*r* = 0.88, *p* < 0.001), and propionate (*r* = 0.89, *p* < 0.001) (Figures 4I, S5I, and S5J). These findings demonstrate that the introduction of microbiota as late as P12 is sufficient to prevent premature ovarian reserve depletion. The correlations between SCFA levels and ovarian gene expression suggest that microbial metabolites may mediate communication between the intestinal tract and ovary.

### SCFAs mediate host transcription in ovarian reserve maintenance

We next tested whether SCFAs alone are sufficient to rescue the ovarian reserve in GF mice. Starting at P12, mirroring the post-natal time point of SCFA availability in colonized mice, GF females received either a SCFA mixture (67.5 mM acetate, 40 mM butyrate, 25.9 mM propionate) or a pH- and sodium-matched solution in their drinking water (Figure 4J). At P70, histological analyses revealed that SCFA supplementation enhanced ovarian morphology and increased the proportion of primordial follicles, while the distribution of growing follicles remained comparable to that of the controls (Figures 4K and 4L). Quantification confirmed higher primordial follicle counts in GF SCFA ovaries compared with GF Na ovaries, with no change in atretic follicle counts (Figures 4M and S14O). The effect was selective for primordial follicles, as the proportions of other follicle types were similar between groups (Figures S5K–S5O). RT-qPCR analysis revealed that SCFA supplementation in GF ovaries also increased the expression of *Nobox* and *Amh*, compared with vehicle-treated GF ovaries (Figures 4N and S5P–S5R). These results demonstrate that microbiota-derived SCFAs influence the expression of genes involved in ovarian reserve maintenance, linking microbial metabolites to ovarian outcomes.

### Dietary fiber protects against high-fat-induced metabolic stress pathways in ovaries

**Figure 5.**
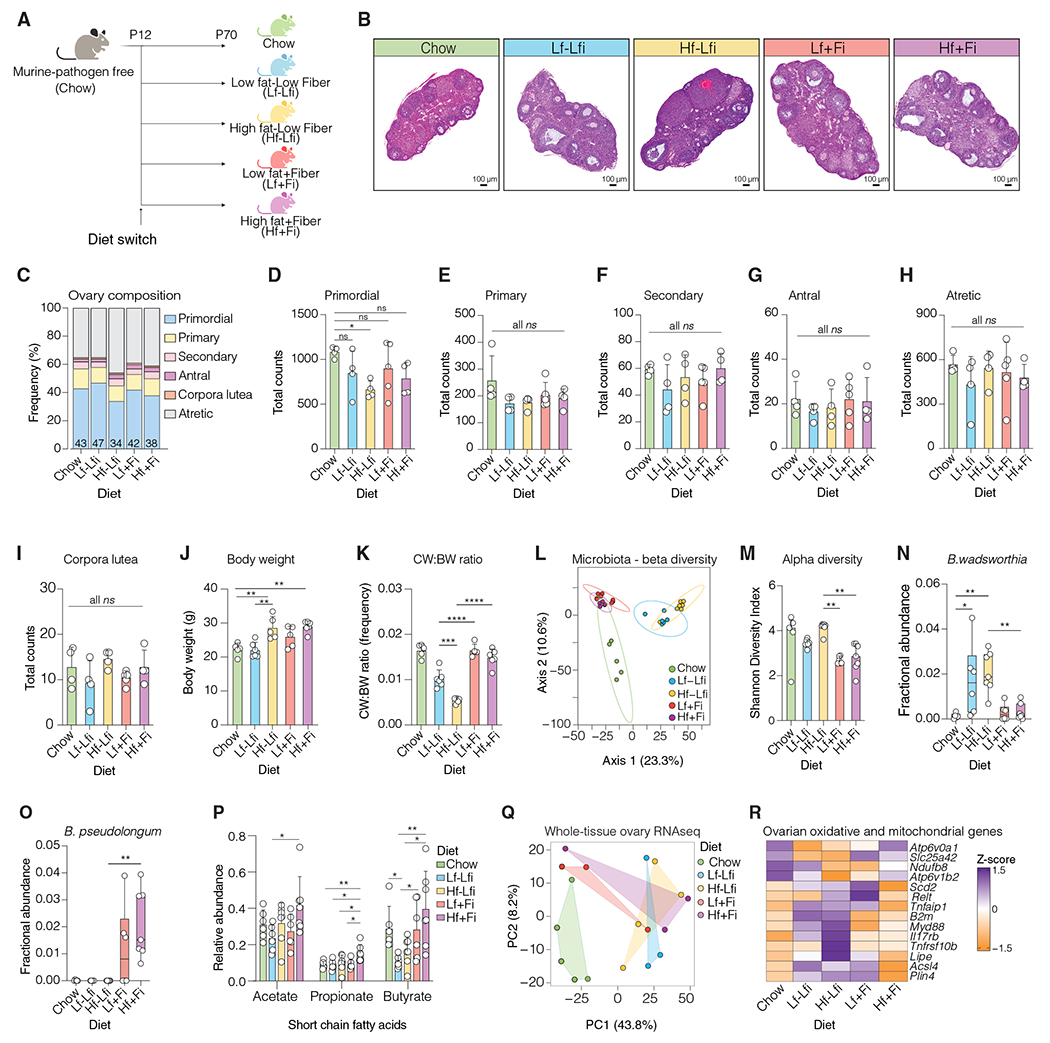
Dietary fiber protects the ovarian reserve through microbiota-dependent mechanisms (A) Experimental design: MPF C57BL/6N female mice were fed either standard chow, Lf-Lfi, Hf-Lfi, Lf+Fi, or Hf+Fi diets from post-natal day 12 (P12) to P70. The composition and ingredient list for diets are in Table S4. Schematic created in BioRender. (B) Representative ovarian histology across dietary groups at P70 (H&E). Scale bars: 100 μM. (C) Ovarian follicle composition across dietary groups. Sample size: chow (*n* = 4), Lf-Lfi (*n* = 4), Hf-Lfi (*n* = 4), Lf+Fi (*n* = 5), Hf+Fi (*n* = 4). (D) Primordial follicle counts across dietary groups. One-way ANOVA with Dunnett’s test relative to chow (*p* < 0.05). Sample size as in (C). (E–I) Quantification of (E) primary, (F) secondary, (G) antral, (H) atretic follicles, and (I) corpora lutea across dietary groups. Sample size as in (C). One-way ANOVA (all ns). (J) P70 body weight across diet groups. Sample size: show (*n* = 5), Lf-Lfi (*n* = 6), Hf-Lfi (*n* = 7), Lf+Fi (*n* = 8), Hf+Fi (*n* = 5). One-way ANOVA with Tukey’s test, **p < 0.01. (K) P70 cecal weight-to-body weight ratio. Sample size as in (J). One-way ANOVA with Tukey’s test, ***p < 0.001, ****p < 0.0001. (L) Principal coordinates analysis of cecal microbiota showing clustering by diet group. Sample size as in (J). PERMANOVA, *p* < 0.001. (M) Alpha diversity of cecal microbiota. Data shown as median ± interquartile range. Sample size as in (J). Kruskal-Wallis test followed by Dunn’s test, **p < 0.01. (N) Fractional abundance of *Bilophila wadsworthia* across diet groups. Sample size as in (J). One-way ANOVA with Tukey’s test. *p < 0.05, **p < 0.01. (O) Fractional abundance of *Bifidobacterium pseudolongum* across diet groups. Sample size as in (J). One-way ANOVA with Tukey’s test, **p < 0.01. (P) Cecal SCFA abundance across diet groups. Sample size as in (J). One-way ANOVA with Tukey’s test, *p < 0.05, **p < 0.01. (Q) Principal-component analysis of whole-tissue ovary RNA-seq data showing primary separation between chow and all custom-formulated diets (PC1, 43.8% variance) with overlapping distributions among refined diets. Sample size: chow (*n* = 5), Lf-Lfi (*n* = 3), Hf-Lfi (*n* = 3), Lf+Fi (*n* = 3), Hf+Fi (*n* = 3). (R) Heatmap showing expression of genes involved in mitochondrial function and oxidative stress response across diet groups. Values represent *Z* scores of normalized expressions. Sample size as in (Q). Data are mean ± SD, unless otherwise noted. MPF, murine pathogen-free; Lf, low fat; Hf, high fat; Lfi, low fiber; Fi, fiber-supplemented. See also Figure S6.

The microbiota weaning transition is triggered by the shift from breast milk to solid foods,^6,67,81^ a process that in mice begins with the transition from breast milk to chow food pellets rich in complex carbohydrates.^82^ While disruptions to diet during this transition have lasting effects on metabolism and immunity, their impact on ovarian outcomes is not well defined.^6,67,81^ To test whether diet at weaning influences reproductive function, we fed MPF females custom-formulated diets from P12 to P70: (1) unrefined grain-based chow diet (serving as a control for comparison to other studies in the literature), (2) low fat-low fiber (Lf-Lfi), (3) high fat-low fiber (Hf-Lfi), (4) low fat+fiber (Lf+Fi), and (5) high fat+fiber (Hf+Fi) diets (Figure 5A). Fiber-supplemented diets contained Hylon VII, a type 2 high-amylose resistant starch that increases SCFA production in rodents and humans.^83–89^ Full macronutrient composition is provided in Table S4. We chose P12 for intervention based on our developmental profiling (Figures 3L–3O, S2, and S3), which identified this stage as the onset of the microbiota transition and as sufficient for rescuing the accelerated loss of ovarian reserve (see Figure 4).

Using this dietary framework, we evaluated how fat and fiber intake from P12 to P70 influenced ovarian follicle dynamics. Histological analysis at P70 revealed morphological differences among diet groups (Figure 5B). Primordial follicles were selectively affected (Figure 5C), with quantification showing a graded reduction across diets. The Hf-Lfi diet led to the most pronounced loss, reducing primordial follicles by 39%, compared with chow-fed mice (662.8 ± 95.8 vs. 1,083 ± 61.3; *p* = 0.0332). Fiber supplementation provided partial protection against high-fat conditions (785.4 ± 182.2; 27% reduction; *p* = 0.1633) (Figure 5D). Even under low-fat conditions, fiber deficiency decreased primordial follicles by 22% (845.8 ± 245.9), whereas fiber supplementation limited this loss to only 17% (897.9 ± 293.9). By contrast, counts of primary, secondary, and antral follicles; corpora lutea; and atretic follicles did not differ across groups (all *p*s > 0.05; Figures 5E–5I). These results show that dietary changes starting at weaning selectively impact the ovarian reserve without altering folliculogenesis or ovulation at this stage.

At P70, body weights were significantly higher in high-fat diet groups regardless of fiber content (Figure 5J). By contrast, cecal weight-to-body weight ratios were lower in the fiber-deficient groups (Lf-Lfi and Hf-Lfi), compared with those in fiber-supplemented groups (Lf+Fi and Hf+Fi) and chow-fed mice (Figure 5K). The combination of high fat and low fiber further suppressed cecal weight-to-body weight ratios, indicating reduced microbial biomass and fermentation activity under these conditions.

To assess how dietary manipulations starting at P12 influence gut microbiota, we performed 16S rRNA gene sequencing on cecal contents from mice at P70. Community structure differed across diets, with principal coordinates analysis showing separation between fiber-supplemented and fiber-deficient groups (Figure 5L). Alpha diversity was lower in the fiber-supplemented groups, compared with Hf-Lfi mice (Figure 5M), consistent with human feeding studies where plant fiber reduces diversity but enhances community stability and metabolic outcomes.^90^ Differential abundance analysis (ALDEx2) revealed a stronger effect of fiber than fat content, with 187 vs. 19 differentially abundant taxa, respectively (Table S5).

Dietary fiber supplementation enriched SCFA-producing families, including *Lachnospiraceae*, *Ruminococcaceae*, and *Bifidobacteriaceae*.^91^ At the species level, *Bilophila wadsworthia*, a sulfate-reducing bacterium linked to diet-induced inflammation, expanded in low-fiber diets regardless of fat content (Figure 5N). *Bifidobacterium pseudolongum*, a SCFA producer with anti-inflammatory effector functions,^91^ increased in fiber-supplemented groups (Figure 5O). These taxonomic shifts corresponded with diet-specific metabolite profiles (Figure 5P). Acetate levels remained consistent across diet groups, except for an increase in Hf+Fi compared with Lf-Fi. Propionate was highest in Hf+Fi compared with all other diets. Butyrate concentrations were higher in fiber-supplemented groups, comparable to chow-fed mice (Figure 5P). These results demonstrate that dietary fiber (e.g., Hylon VII) shapes gut microbial composition and metabolic output independently of fat content, consistent with prior work showing that microbiota composition can be uncoupled from body weight in response to diet.^92,93^

To investigate the molecular basis of dietary effects on follicle dynamics, we performed RNA-seq analysis on P70 ovaries from each group. Principal-component analysis showed primary separation between chow and all refined diets along PC1 (43.8% of variation), whereas the refined diets overlapped (Figure 5Q). Differential expression analysis revealed extensive differences between refined diets and chow, with 4,529 DEGs in Hf−Lfi vs. chow, 7,159 DEGs in Hf+Fi vs. chow, 5,283 DEGs in Lf−Lfi vs. chow, and 4,590 DEGs in Lf+Fi vs. chow (FDR < 0.25) (Table S6). In contrast, few genes distinguished high- vs. low-fat diets under low-fiber conditions (9 DEGs), whereas fiber-supplemented diets showed 8,690 DEGs between high- and low-fat groups.

Given the effects of diet on primordial follicle counts, we next examined expression of genes critical for maintaining ovarian reserve (*Nobox*, *Foxo3*, *Figla*, *Bmp15*, *Foxl2*, *Gdf9*, *Amh*, *Kit*). Gene expression patterns paralleled histological findings. In low-fat settings, fiber supplementation preserved expression at chow-like levels, consistent with minimal follicle loss. Under high-fat conditions, these genes remained downregulated despite fiber supplementation (except for *Kit* and *Inhba*), mirroring follicle quantifications (Figures 5D and S6).

Pathway enrichment analysis showed that the protective effects of dietary fiber in a high-fat environment extend beyond ovarian reserve genes. In Hf+Fi ovaries, pathways disrupted in Hf−Lfi ovaries involved in inflammation, metabolism, and oxidative stress response were normalized. Energy metabolism and mitochondrial function were the most significantly enriched categories, accounting for 32.5% and 27.1% of all overrepresented pathways, respectively (Table S6). Other enriched categories included apoptosis/survival (13.3%), epigenetic regulation (13.0%), and oxidative stress response (11.8%). Fiber normalized several pathways disrupted by the Hf−Lfi diet, including mitochondrial respiratory chain complex assembly (FDR = 0.00078 in Hf−Lfi vs. chow, FDR = 0.31247 in Hf+Fi vs. chow), oxidative phosphorylation (FDR = 0.00254 in Hf−Lfi vs. chow, FDR = 0.29144 in Hf+Fi vs. chow), cellular response to oxidative stress (FDR = 0.00467 in Hf−Lfi vs. chow, FDR = 0.25863 in Hf+Fi vs. chow), and regulation of inflammatory response (FDR = 0.00623 in Hf−Lfi vs. chow, FDR = 0.28976 in Hf+Fi vs. chow) (Table S6). These fiber-dependent improvements align with known mechanisms of high-fat diet-induced oocyte damage, where mitochondrial dysfunction, oxidative stress, and inflammation reduce oocyte quality and developmental potential in mice and humans (Figure 5R).^27,94–98^

### Dietary fiber protects oocyte quality and embryo competence in conditions of a high-fat diet

**Figure 6.**
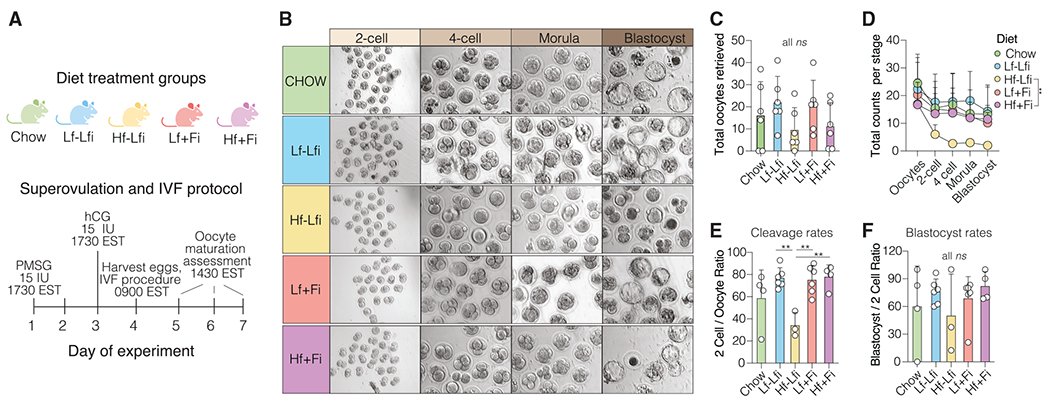
Microbiota-targeted dietary intervention preserves reproductive competence (A) Experiment for superovulation and IVF: P70 females received pregnant mare serum gonadotropin (PMSG) (15 IU) on day 1, followed by human chorionic gonadotropin (hCG) (15 IU) on day 3. Oocytes were harvested, and IVF was performed on day 4, with embryo development assessed at 24-h intervals. Schematic created in BioRender. (B) Representative images of embryo development at 2-cell, 4-cell, morula, and blastocyst stages following IVF. (C) Total oocytes retrieved following superovulation across diet groups. Sample size: chow (*n* = 6), Lf-Lfi (*n* = 6), Hf-Lfi (*n* = 6), Lf+Fi (*n* = 6), Hf+Fi (*n* = 6). One-way ANOVA (ns, not significant). (D) Quantification of embryo development across successive stages following IVF. Two-way ANOVA with Tukey’s test. Sample sizes: chow (*n* = 4), Lf-Lfi (*n* = 6), Hf-Lfi (*n* = 3), Lf+Fi (*n* = 6), Hf+Fi (*n* = 4). Mice yielding ≥5 oocytes upon superovulation from (C) were included in this analysis to ensure that the assessment of embryo competence was not confounded by a poor superovulation response. (E) Cleavage rates (2-cell/oocyte ratio) following IVF. Sample sizes as in (D). One-way ANOVA with Tukey’s test, **p < 0.01, ***p < 0.001. (F) Blastocyst development rates (blastocyst/2-cell ratio) following IVF. Sample sizes as in (D). One-way ANOVA with Tukey’s test, ns, not significant. Data are mean ± SD. MPF, murine pathogen-free; Lf, low fat; Hf, high fat; Lfi, low fiber; Fi, fiber-supplemented.

We next asked whether normalization of transcriptional pathways by dietary fiber leads to better reproductive outcomes. This question is clinically relevant, as obesity reduces pregnancy success and *in vitro* fertilization (IVF) success rates by 30%–50% in women, largely due to early embryo cleavage failure.^99–101^ To test this, we performed superovulation and IVF at P70 in all diet groups (Figure 6A). Morphological analysis revealed impaired development in Hf−Lfi embryos, including poor compaction, irregular cell arrangements, and defective blastocoel formation (Figure 6B). Oocyte yields after superovulation were similar across groups (Figure 6C). Counts of embryos at each developmental stage revealed that Hf−Lfi embryos declined starting at the 2-cell stage, whereas the other groups progressed (Figure 6D). Cleavage rates (calculated as the 2-cell/oocyte ratio) were significantly lower in Hf−Lfi embryos (~30%) compared with ~70%–80% in all other diets, mirroring early embryo defects observed in obese women undergoing IVF.^102–104^ Fiber supplementation rescued cleavage efficiency in high-fat mice (Figure 6E). Blastocyst formation followed similar trends but did not reach statistical significance (Figure 6F), suggesting that the primary defect lies in oocyte quality rather than post-fertilization development, a pattern also seen in human obesity-related subfertility.^105,106^ These findings show that dietary fiber protects against high-fat diet-induced impairments in oocyte quality and early embryo competence by normalizing transcriptional pathways involved in inflammation, mitochondrial function, and oxidative stress response.

## DISCUSSION

The preservation of germ cells is crucial for species survival. We demonstrate that the microbiota extends the reproductive lifespan of mice by regulating primordial follicle loss. In the absence of the microbiota, accelerated activation, impaired progression, and increased atresia prematurely deplete the ovarian reserve, a phenotype rescued by colonization or SCFA supplementation. Dietary fiber protects oocyte quality and embryo competence even under high-fat conditions. These findings provide a mechanistic explanation for the long-observed reduced reproductive capacity of GF mice and broaden the scope of host-microbe communication to include ovarian function.

Our data identify the post-natal period as a crucial window for microbiota-dependent ovarian regulation. This period aligns with the expansion of SCFA-producing bacteria during the weaning transition. Disruption of this process affects ovarian follicle dynamics, beginning with altered gene expression patterns and culminating in changes in follicle counts. Colonization restored gene expression patterns without increasing the number of growing follicles and normalized genes spanning multiple cell types beyond oocytes. These findings suggest that the microbiota influences ovarian gene expression through multiple mechanisms, both by affecting the rate of follicle loss and by directly impacting non-oocyte cell populations and metabolic pathways within the ovary.

SCFAs were identified as potential mediators based on colonization experiments showing correlations between cecal SCFA availability and the expression of genes involved in maintaining ovarian reserve. While direct SCFA supplementation rescued primordial follicle counts and restored *Amh* expression, it showed smaller effects than complete colonization and failed to normalize other key genes, such as *Kit* and *Gdf9*. This suggests that microbiota influences ovarian function through both SCFA-dependent and -independent mechanisms, with future work poised to uncover these additional pathways.

As dietary fiber influences the microbial production of SCFAs, we sought to determine whether manipulation of dietary fat and fiber during this early life period could affect reproductive outcomes in adulthood. Fiber supplementation affected SCFA-producing bacteria abundance, increased SCFA availability, and protected reproductive outcomes in conditions of a high-fat diet. Fiber supplementation reversed high-fat diet-induced impairment of embryo competence, increasing cleavage rates from 30% to 70%–80%. This protection was linked to inflammatory, metabolic, and oxidative stress response pathways in ovarian tissue, aligned with known lipotoxicity pathways in reproductive tissues, where elevated free fatty acids disrupt mitochondrial function, increase oxidative stress, and alter cellular metabolism in oocytes.^98^ In this context, key functions of SCFAs include modulation of the mitochondrial respiratory chain activity^106^ and inflammation,^107,108^ suggesting a link between dietary fiber-derived metabolites and reproductive outcomes, which warrants further investigation.

The relationship between diet, microbiota, and fertility outcomes offers a potential explanation for increasing infertility rates in populations consuming Western diets and those undergoing demographic transitions.^26,29,107–109^ Alterations to the gut microbiota in women with primary ovarian insufficiency and polycystic ovarian syndrome further support the clinical significance of our findings.^24,110–113^ Future studies should examine the relationship between diet, microbiota, and ovarian reserve in individuals with reproductive disorders and assess the efficacy of microbiota-directed interventions in improving fertility outcomes.

### Limitations of the study

GF models provide mechanistic insights, but they represent artificial conditions not found in nature, which limits their translational relevance. Our dietary intervention experiments analyzed the microbiome only at P70, not during the critical P12–P28 window when we hypothesize ovarian programming occurs. Although dietary manipulations starting at P12 had lasting effects, we lack direct evidence of microbial changes during the weaning transition.

We did not validate SCFA-dependent mechanisms using global or conditional knockout models, nor did we identify specific SCFAs mediating the observed effects. Our continuous dietary intervention design (P12–P70) cannot determine whether early treatments maintain ovarian follicle counts or whether later interventions improve oocyte quality, which has significant therapeutic implications. The optimal timing to achieve the most beneficial effects remains unknown.

The specific molecular mechanisms connecting SCFAs to ovarian gene expression remain unresolved. While our focus was on SCFAs, comprehensive metabolomics will identify additional microbial mediators that affect ovarian function. Our work cannot draw conclusions about whether the crosstalk between the microbiota, their metabolites, and ovarian tissue observed in mice is similar in humans, as significant differences exist in diet, microbiota composition, and reproductive physiology. This presents an exciting area for future research to explore.

## RESOURCE AVAILABILITY

### Lead contact

Further information and requests should be directed to and will be fulfilled by the lead contact, Eldin Jašarević (eldin.jasarevic@pitt.edu).

### Materials availability

This study did not generate new reagents, and all reagents were commercially purchased and used as received.

## STAR★METHODS

### EXPERIMENTAL MODEL AND STUDY PARTICIPANT DETAILS

All experiments were approved by the University of Pittsburgh and Magee-Womens Research Institute Institutional Animal Care and Use Committee and performed in accordance with the National Institutes of Health Animal Care and Use Guidelines. Murine-pathogen-free (MPF) C57Bl/6N female and male mice were purchased from Taconic Biosciences (C57Bl/6NTac). All mice were maintained on a 12-hour light/dark cycle (lights on 0600 EST; lights off 1800 EST). The room temperature was maintained between 75-77°F and 40-60% humidity. An Onset HOBO MX2202 Wireless Temperature/Light Data Logger (HOBO Data Loggers, Wilmington, NC) was used to confirm light-dark photoperiod stability. Unless otherwise specified, ad libitum access was provided to water and a grain-based rodent chow diet (NIH-31M Rodent Diet, Envigo; 20.940% protein, 65.324% carbohydrate, 13.736% fat). Germ-free C57Bl/6NTac female and male mice were purchased from Taconic Biosciences and housed in the University of Pittsburgh Gnotobiotic Facility, with ad libitum access to water and the same grain-based rodent chow diet (NIH-31M Rodent Diet, Envigo; 20.940% protein, 65.324% carbohydrate, 13.736% fat).

For diet manipulation studies, custom diets were created in partnership with Teklad Envigo to isolate the effects of dietary fat and fiber levels. Starting at postnatal day 12 (P12), female mice had free access to one of the following diets: (1) low fat-low fiber diet (Lf−Lfi; 20% protein, 70% carbohydrate, 10% fat); (2) low fat with fiber diet (Lf+Fi; 20% protein, 65% carbohydrate, 10% fat, containing 5% Hylon VII resistant starch); (3) high fat-low fiber diet (Hf−Lfi; 20% protein, 35% carbohydrate, 45% fat); (4) high-fat with fiber diet (Hf+Fi; 20% protein, 30% carbohydrate, 45% fat, with 5% Hylon VII resistant starch); or (5) standard grain-based chow diet (Chow; NIH-31M, as described above). The P12 time point was chosen because it marks a key developmental period for microbiota-dependent ovarian programming. Hylon VII, a type 2 high-amylose resistant corn starch, served as the fiber source based on extensive literature demonstrating its ability to boost short-chain fatty acid production in rodents and humans. The complete macronutrient details and ingredient list for custom diets are provided in Table S4. All diets used in the experiments were irradiated. All experiments, except for breeding necessary to produce offspring, were conducted on female mice.

### METHOD DETAILS

#### Breeding information for analyses of lifetime breeding records

Taconic Biosciences maintained the C57Bl/6N Tac mice used in lifetime reproduction analyses under standardized breeding protocols. C57Bl/6N mating pairs begin breeding at 8-10 weeks of age and continue until they either produce 9 litters or reach 32 weeks of age. Males stay housed with females throughout the breeding period. The average litter interval for conventional pathogen-free C57Bl/6N Tac mice is 21-28 days, with a typical gestation of 19-21 days. Females experience postpartum estrus, allowing them to conceive immediately after delivery. Strain-specific litter sizes for MPF mice typically range from 6 to 12 pups and can vary based on parity. Pups are weaned at 21 days old. For our analysis of lifetime breeding records, we collected data from 493 litters of conventionally raised MPF and GF C57Bl/6N Tac mice, which were maintained under consistent breeding conditions, allowing for a reliable comparison of reproductive outcomes between these groups.

#### Confirmation of germ-free status

The University of Pittsburgh Gnotobiotic Facility conducts quarterly screenings for bacterial contamination in germ-free mice. Screening for bacterial contamination in mouse feces involves both aerobic and anaerobic bacterial cultures, as well as quantitative real-time PCR (qRT-PCR) assays. Tests were negative on all assays from isolators housing the germ-free mice used in this study before the start of the experiments and after their completion. This verified the germ-free status of the mice used in these studies.

#### Germ-free mice microbial colonization

Two independent cohorts of time-mated, germ-free pregnant dams were housed in four separate germ-free isolators during pregnancy. Each isolator was randomly assigned to one of two groups: 1) pregnant dams that were conventionalized the afternoon before giving birth (designated as Ex-GF P0); 2) lactating dams that were colonized on day 12 post-delivery (designated as Ex-GF P12). For the microbial gavage preparation, ileal and cecal luminal contents were collected from four 8-week-old, murine pathogen-free C57Bl/6N Tac female mice. The contents were weighed, transferred to a sterile tube containing Rosshart Freezing Medium (RFM),^114,115^ and homogenized. Final gavage aliquots were prepared at a 1:30 dilution (material to RFM) and used for administering the microbiota. Female mice received a 200 μL oral gavage of this preparation. Fecal pellets were collected from colonized mice at two time points: one day before gavage and seven days afterward.

#### Short-chain fatty acid (SCFA) reconstitution

SCFA reconstitution experiments were conducted in one germ-free isolator. Time-mated germ-free dams were allowed to deliver, and 12 days after delivery, the cages were randomized to receive drinking water supplemented with either a SCFA mix (67.5 mM sodium acetate, 40 mM sodium butyrate, 25.9 mM sodium propionate) or a pH- and salt-balanced vehicle control, following a previously published protocol.^116^ The supplemented water was prepared fresh once a week, and the drinking water was replaced weekly. Offspring remained in their respective water treatment groups after weaning and until postnatal day 70, when tissue collections were conducted.

#### Tissue processing and Histological staining

Mouse ovaries were fixed in 4% paraformaldehyde (Thermo Scientific) at 4° C overnight. The tissue was then washed multiple times in calcium- and magnesium-free PBS (Lonza). Washed tissues were embedded in paraffin, and 5 μm sections were cut through the entire ovary. Sections were mounted onto Superfrost-plus slides, and slides were stained with Hematoxylin-eosin or Periodic Acid-Schiff (PAS). The Magee-Women’s Research Institute histology and microimaging core performed all tissue processing. Masson’s Trichrome staining was performed according to the manufacturer’s instructions using the Masson’s Trichrome Stain Kit (Polysciences cat #25088)

#### Microscopy

Image analysis was conducted using Nikon Elements AR software on the Nikon 90i fully motorized imaging platform. Post-imaging analysis was performed using ImageJ version 2.14 software.

#### Quantification of ovarian follicles and mathematical modeling

Follicle counts started from the first section, examining ovarian tissue every 10th section of serially sectioned tissue throughout the entire ovary.^38,39,55,56,117–121^ Primordial follicles were classified as oocytes with a single layer of squamous granulosa cells. Primary follicles contained an oocyte surrounded by cuboidal granulosa cells. Oocytes partially surrounded by both squamous and cuboidal granulosa cells were also counted as primary follicles. Secondary follicles contain an oocyte with more than one layer of granulosa cells. Antral follicles feature oocytes surrounded by multiple layers of granulosa and theca cells, along with a fluid-filled antrum. The corpus luteum contains granulosa lutein cells with the characteristic appearance of steroid-producing cells, characterized by pale cytoplasm, as well as smaller, more deeply stained theca lutein cells.

Atretic follicles were identified by a deeply stained zona pellucida that was detached from the granulosa cells, with or without a fragmented oocyte and somatic cells, which may have pyknotic nuclei. For the atretic follicles counts, we used both H&E and PAS staining. PAS staining highlights the basement membrane and other glycoproteins in the ovarian stroma, allowing for a clear distinction between healthy follicles and atretic ones. It also confirmed the presence of zona pellucida protein on atretic follicles. This distinction is vital because atretic follicles can be miscounted when relying only on H&E staining, due to its limitations in showing these features. Combining PAS with H&E ensures accurate identification.

To prevent double-counting, only follicles containing an oocyte nucleus were counted. Three blinded, independent scorers quantified follicles across all studies, and inter-rater reliability was confirmed. The number of follicles was determined by applying both the sampling frequency (10) and a correction factor to estimate the total number of follicles in a whole ovary, then multiplying by 2 to estimate the total number of follicles in the entire animal, as previously described.^38,39,55,56,117–121^ The correction factor was calculated as follows: (section thickness (5μm) ÷ (section thickness (5μm) + average follicle nucleus diameter)). Oocyte nuclear diameters were measured in at least 10 representative follicles from each animal using ImageJ version 2.14 software.^56^

#### Mathematical modeling of ovarian follicle dynamics ∫

To characterize patterns of follicle loss and atresia accumulation, we fitted multiple mathematical models (linear, exponential, and power functions) to follicle count data across developmental time points for both MPF and GF groups.^39,60–63^ Statistical model comparison utilized the Akaike Information Criterion (AIC), with the model having the lowest AIC value being considered the most parsimonious model.^122^ R^2^ values were calculated to assess goodness of fit. For each model type, we used the following equations:

(Equation 1) linear model: y=intercept+slope×x

(Equation 2) exponential model: y=a×exp(−b×x)

(Equation 3) $$\text{power law function model: y}=\text{a}\times {\text{x}}^{\text{b}}+\text{c}$$

A compartmental analysis of follicle dynamics was conducted using a six-parameter model that describes the transition rates between follicle stages. This model is based on the following biological assumptions: (1) follicles progress unidirectionally through discrete developmental stages; (2) transitions between stages can be modeled by first-order kinetics, where the transition rate is proportional to the number of follicles in each stage; (3) transition rates stay relatively constant within specific developmental windows; and (4) follicles at the same stage have an equal probability of moving to the next stage or becoming atretic. While this simplifies the complex biology of folliculogenesis, these assumptions are supported by previous mathematical models of follicle dynamics64. The six transition rates calculated were: primordial follicle activation (k1), primary-to-secondary progression (K2), primary follicle atresia (k3), secondary-to-antral progression (k4), secondary follicle atresia (k5), and antral follicle atresia (k6). Transition rates were determined between adjacent timepoints by comparing net changes in follicle counts at each stage. To validate our compartmental model results, we conducted an additional direct analysis of stage-to-stage ratios, calculating efficiency metrics: activation (Primary/Primordial), primary progression (Secondary/Primary), secondary progression (Antral/Secondary), and atresia tendency (Atretic/Total). These efficiency ratios offer a model-independent evaluation of follicle dynamics. All modeling and statistical analyses were performed using R (version 4.2.1) with tidyverse, minpack.lm, and patchwork packages within the R environment.^123^

#### Superovulation and in vitro fertilization

Female mice from the Chow, Lf−Lfi, Hf−Lfi, Lf+Fi, and Hf+Fi groups were injected intraperitoneally (IP) with 15 IU of pregnant mare serum gonadotropin (PMSG) (ProSpec #hor-272) at 5:30 PM on Day 1. On Day 3, forty-eight hours after PMSG, the mice received an IP injection of 15 IU of human chorionic gonadotropin (hCG) (Sigma #CG5). On Day 4, at 9:00 AM, female mice were sacrificed, and their oviducts were removed. Cumulus oocyte complexes (COCs) were collected from the ampulla of the oviduct, and the oocytes were then released from the COCs in M2 medium (Sigma-Aldrich) containing 0.1% hyaluronidase. The oocytes were washed in M2 medium and then placed in 200 μl of CARD medium (Cosmo Bio USA #KYD-003-EX) and incubated in a humidified chamber at 37° C with 5% CO2. A 10-week-old C57BL/6 male mouse on a Chow diet was sacrificed by cervical dislocation. Cauda epididymal sperm were collected and capacitated in 200 μl of CARD FERTIUP preincubation Medium (Cosmo Bio USA) in an incubator at 37° C with 5% CO2 for 60 minutes. Eight microliters (8 μL) of sperm were added to each drop of CARD medium containing oocytes and incubated at 37° C with 5% CO2 for at least 6 hours. Oocytes were washed three times in 80 μl drops of KSOM (Sigma), then transferred to a fresh drop of KSOM covered with liquid paraffin and cultured in the incubator (37° C, 5% CO2 in air) until the blastocyst stage was reached. Embryo development was assessed daily from days 5to 8 post-fertilization. Samples yielding fewer than five oocytes were excluded from embryo competence evaluation, and this criterion was applied uniformly across all treatment groups to ensure that only mice responding to superovulation were included in the study. For statistical purposes, oocytes from each female were processed and cultured separately. Cells were counted with the Nikon SMZ 645 Zoom stereomicroscope. Images were captured with the Nikon Eclipse Ti microscope and Nikon digital sight camera.

#### 16S rRNA marker gene sequencing and data analysis

Genomic DNA was extracted from 50 mg of cecal luminal contents using the MagAttract PowerMicrobiome DNA/RNA Kit (Qiagen), with bead-beating on a TissueLyser II (Qiagen) according to the manufacturer’s instructions. 16S libraries were generated using a two-step PCR protocol. Amplicon PCR was performed as follows for amplification of the 16S rRNA V3-V4 region from cecal luminal contents: initial denaturation at 95° C for 3 minutes, followed by 25 cycles of 95° C for 30 seconds, 55° C for 30 seconds, 72° C for 30 seconds, and a final extension at 72° C for 5 minutes. Resultant 16S V3-V4 amplicons were then purified using AMPure XP beads at a 0.8 ratio of beads to amplicon volume. Illumina Nextera XT v2 Index Primer 1 (N7xx) and Nextera XT v2 Index Primer 2 (S5xx) were index primers. Index PCR was performed as follows for amplification of the 16S rRNA V3-V4 region from cecal luminal contents: initial denaturation at 95° C for 3 minutes, followed by eight cycles of 95° C for 30 seconds, 55° C for 30 seconds, 72° C for 30 seconds, and a final extension at 72° C for 5 minutes. Indexed libraries were cleaned up using AMPure XP beads at a 0.8 ratio of beads to the indexed library. The concentration of indexed libraries was quantified using a Qubit 4 fluorimeter, and library fragment size was quantified using an Agilent Tapestation 4200 with D5000 ScreenTapes. Normalized and pooled libraries were verified for even read coverage using an Illumina iSeq 100 before being sequenced on a NextSeq 2000 using 2x300 bp PE chemistry at the Health Sciences Sequencing Core at Children’s Hospital of Pittsburgh.

Sequences were demultiplexed using the BaseSpace Sequence Hub with bcl2fastq2 conversion software (version 2.2.0). Raw sequences underwent quality filtering with a minimum Phred quality score of 20 and were trimmed to remove low-quality regions. Chimeric sequences were identified and removed using the consensus method. ASVs were generated using the DADA2 denoise-single function,^124^ trimming 33 bp of the primer sequence and truncating reads at positions 280 (forward) and 250 (reverse) based on quality profiles. Taxonomic classification was performed using a Naive Bayes classifier trained on the SILVA reference database^125^ (version 138) with the q2-feature-classifier. A phylogenetic tree was constructed using the MAFFT alignment^126^ and FastTree2^127^ methods to facilitate phylogenetic diversity analyses. Feature tables containing ASV counts per sample and corresponding taxonomy were exported for downstream analyses.

Quality-filtered sequences were processed with phyloseq^128^ (version 1.40.0) for downstream analyses. Potential contaminants were identified and removed using the decontam package^129^ (version 1.16.0) by analyzing prevalence patterns in negative controls versus actual samples. Non-target sequences,^130^ including *Cyanobacteria* and chloroplasts, *Rickettsiales*, mitochondria, and *Lactococcus* (a common contaminant in refined diet studies^131^), were removed. Low-abundance ASVs (less than 10 total reads across all samples) and rare ASVs (present in <5% of samples) were filtered out. The quality filtering pipeline retained approximately 95% of total sequence reads while reducing the ASV count by approximately 60-70%, ensuring robust and reliable downstream analyses.

For each experiment (e.g., development, colonization, and diet manipulation), data were analyzed independently after filtering to include only ASVs present in the relevant samples. Sequencing yielded an average of 27,611 reads per sample (range: 10,939-42,407). For alpha diversity analyses, samples were rarefied to the minimum sequencing depth within each experimental group: development/weaning (5,618 reads), colonization (4,511 reads), and diet experiment (6,793 reads). Taxonomic composition was analyzed at both phylum and genus levels. Alpha diversity was evaluated using observed ASV richness (Observed Counts) and Shannon diversity indices, and statistical comparisons between groups were performed using Kruskal-Wallis tests with post-hoc Dunn’s test for multiple comparisons. Beta diversity was assessed using Aitchison distance (Euclidean distance on center log ratio (CLR)-transformed data) to handle the compositional nature of 16S rRNA marker gene profiling datasets. Statistical significance of group differences was assessed using permutational multivariate analysis of variance (PERMANOVA) with 999 permutations.

Differential abundance testing was performed using ALDEx2^65^ (ANOVA-Like Differential Expression tool for compositional data, version 1.28.0) with Welch’s t-test and Benjamini-Hochberg FDR correction. This method employs a center log-ratio transformation and Monte Carlo sampling from a Dirichlet distribution to accurately account for the compositional nature of microbiome data. Features with an FDR < 0.25 were considered differentially abundant between groups. For the colonization experiment, samples were grouped by colonization time point (Early: P0; Late: P12) and age at collection (P28, P70), allowing assessment of colonization timing effects on microbiota composition. For the development experiment, samples from P7, P12, P21, P28, and P70 were compared to characterize microbiota succession patterns in MPF female mice. For the diet experiment, samples were grouped by dietary conditions (Chow, low-fat low-fiber [Lf−Lfi], low-fat with fiber [Lf+Fi], high-fat low-fiber [Hf−Lfi], and high-fat with fiber [Hf+Fi]) to test the effects of dietary fat and fiber content on microbiota composition. We specifically focused on the main effects of fiber (high-fiber vs. low-fiber) and fat (high-fat vs. low-fat), as well as individual comparisons of each diet group to Chow. For species-level analysis, we extracted ASVs (e.g., *Bilophila*, *Desulfovibrio*, *Faecalibaculum rodentium*, *Bifidobacterium*, and *Roseburia*) from the ALDEx2 results across all diet comparisons. We confirmed species-level annotation using NCBI BLAST for the ASV sequence using rRNA search function.

#### Whole shotgun metagenomics and data analysis

Genomic DNA extracted for the 16S rRNA marker gene sequencing was utilized. Shotgun metagenomic libraries were prepared using the Illumina DNA prep kits according to the manufacturer’s protocol. The quality and quantity of the libraries were assessed using Qubit fluorometric quantitation and Agilent Tapestation 4200 with D5000 ScreenTapes. Sequencing was performed on an Illumina NextSeq 2000 platform, using 2 x 300 bp paired-end sequencing. A target sequencing depth of 40 million reads per sample was aimed for to ensure adequate coverage of the metagenomes. Raw sequencing data were processed using the WGSA2 (Whole Genome Shotgun Analysis 2) pipeline available on the Nephele platform (https://nephele.niaid.nih.gov/pipeline_details/wgsa/).^132^ The pipeline was executed with default parameters unless otherwise specified. For quality control and preprocessing, raw reads were trimmed and filtered for quality using Trimmomatic.^133^ For host DNA removal, sequences matching the mouse genome were removed using Bowtie2.^134^ Filtered reads were classified using Kraken2,^135^ and protein-coding sequences were predicted and annotated using Prodigal^136^ and compared against the KEGG database.^137,138^

#### Quantification of 3NP-Short Chain Fatty Acids

Quantification of 3NP-Short Chain Fatty Acids was performed at the University of Pittsburgh Health Sciences Mass Spectrometry Core. Cecal luminal contents and ovarian tissue samples were homogenized with 50% aqueous acetonitrile at a ratio of 1:15 vol: wt.^139^ Deuterated internal standards (5 μg/mL): formate-d2, acetate-d4, butyrate-d5, proprionate-d6, valerate-d2, and hexanoate-d4 (CDN Isotopes, Quebec, Canada) were added. Samples were homogenized using a FastPrep-24 system (MP-Bio), with Matrix D at 60 Hz for 30 seconds, before being cleared of protein by centrifugation at 16,000xg. Plasma samples were cleared of protein using 4x volumes of ice-cold 1:1 MeOH: EtOH with vortexing, followed by centrifugation at 16,000xg. Cleared supernatant (60 μL) was collected and derivatized using 3-nitrophenylhydrazine (3-NP). Each sample was mixed with 20 μL of 200 mM 3-nitrophenylhydrazine in 50% aqueous acetonitrile and 20 μL of 120 mM N-(3-dimethylaminopropyl)-N‘-ethyl carbodiimide 6% pyridine solution in 50% aqueous acetonitrile. The mixture was reacted at 60° C for 40 minutes, and the reaction was stopped with 0.45 mL of 50% acetonitrile. Absolute quantitation of SCFAs was performed by preparing 9-point calibration curves for formate (1.102 fmol/μL to 0.241 nmol/μL) acetate (0.8 fmol/μL to 0.185 nmol/μL), propionate (0.6 fmol/μL to 0.150 nmol/μL), and butyrate (0.5fmol/μL to 0.126 nmol/μL) and were derivatized as described above. Derivatized samples were injected (5 μL) via a Thermo Vanquish UHPLC and separated over a reversed-phase Phenomenex Kinetex 150 mm x 2.1 mm 1.7 μM particle C18 maintained at 55° C. For the 20-minute LC gradient, the mobile phase consisted of solvent A (water/0.1% FA) and solvent B (acetonitrile/0.1% FA). The gradient started at 15% B for 2 minutes, increased to 60% B over 10 minutes, and then to 100% B over 1 minute. The gradient was held at 100% B for 3 minutes, before equilibration at 15% B for 4 minutes. The Exploris 240 hybrid mass spectrometer was operated in positive ion mode, scanning in ddMS2 mode (2 μscans) from 75 to 1000 m/z at 120,000 resolutions with an AGC target of 2e5 for full scan, 2e4 for MS2 scans using HCD fragmentation at stepped 15, 35, 50 collision energies. The source ionization setting was 3.0 kV spray voltage for positive mode. Source gas parameters were 45 sheath gas, 12 auxiliary gas at 320° C, and 3 sweep gas. Calibration was performed before analysis using the PierceTM FlexMix Ion Calibration Solutions (Thermo Fisher Scientific). Integrated peak areas were then extracted manually using Quan Browser (Thermo Fisher Xcalibur ver. 2.7) for both SCFA and organic acids.^140^ SCFAs are reported as the area ratio of SCFA to the internal standard before conversion to absolute concentration. Organic acid peak areas were normalized to the butyrate-d5 internal standard and reported as Relative Abundance.

#### Whole-tissue RNA sequencing and data analysis

Frozen tissue samples were homogenized in QIAzol Reagent (Qiagen) using a MiltenyiBiotec gentleMACS Octo Dissociator for 30s. RNA was isolated with Qiagen RNeasy Mini Kits according to the manufacturer’s instructions. RNA integrity was quantified on an Agilent TapeStation 4200 using TapeStation RNA ScreenTapes. All samples had an RIN score above 8. Sequencing libraries were prepared using Illumina Stranded mRNA prep, Ligation kits with IDT for Illumina RNA UD Indexes Set A, and Ligation index adapters. The concentration of indexed libraries was quantified using Qubit, and library fragment size was quantified using an Agilent Tapestation 4200 with D5000 ScreenTapes. Sequencing was performed on an Illumina NextSeq 2000 using P3 flow cells and 2x100 paired-end run geometry at the Health Sciences Sequencing Core at Children’s Hospital of Pittsburgh. Sequencing yielded an average of 30–60 million reads per sample. Concatenated FASTQ files were processed through a quality control pipeline using FastQC^141^ (v0.11.9) and MultiQC^142^ (v1.11) to assess read quality, adapter content, and duplication rates. High-quality reads were pseudo-aligned to the Mus musculus reference transcriptome (Ensembl version 101, GRCm38) using kallisto^143^ (v0.46.1) with parameters “–bias –rf-stranded –bootstrap-samples=60” to account for technical biases and improve quantification accuracy. Transcript-level abundance estimates were collapsed to gene-level using tximport153 (v1.20.0) with the “lengthScaledTPM” option to correct for transcript length biases when summarizing to gene-level counts. Quality-filtered gene count matrices were analyzed using a robust statistical pipeline. Genes with low expression (counts per million <1 in at least 3 samples) were filtered. Counts were normalized using trimmed mean of M-values (TMM) normalization in edgeR^144^ (v3.34.1) to account for library size differences and RNA composition biases. Mean-variance relationships were modeled using voom^145^ (limma v3.48.3) to transform count data to log2-counts per million (log2 CPM) with associated precision weights. Differential gene expression analysis was conducted using linear modeling with empirical Bayes moderation of standard errors^145^ (limma v3.48.3). Multiple testing correction used the Benjamini-Hochberg procedure to control FDR at 0.25. Heatmaps were visualized using ggplots^146^ and heatmaply.^147^

#### Analysis of microbiota status on developmental gene expression

To investigate the temporal dynamics of molecular changes during ovarian development, we performed differential gene expression analysis between GF and MPF ovaries at each developmental timepoint (P7, P12, P21, P28, and P70) as described above. For each time point, genes were considered differentially expressed at FDR < 0.25. Principal component analysis was performed to visualize the global transcriptional differences between GF and MPF samples across developmental timepoints. Age-specific pathway enrichment analysis was conducted to identify biological processes uniquely affected at each developmental stage. Differentially expressed genes at each time point were subjected to GO and KEGG pathway analysis using clusterProfiler^148,149^ (v3.18.1). Developmental stage-specific pathway signatures were identified by comparing enriched pathways across timepoints and extracting those uniquely or predominantly enriched at specific ages.

For integrated pathway-level analysis of microbiota effects, we used gene sets of key ovarian regulatory processes based on literature evidence and gene ontology annotations. Each pathway was validated using multiple independent sources to ensure comprehensive coverage (see references in Table S2): follicle activation (35 genes including *Foxo3*, *Pten*, *Nobox*, *Kit/Kitl*, *Amh/Amhr2*), follicle progression (50 genes including *Fshr*, *Gdf9*, *Bmp15*, *Star*, *Esr1*, *Igf1/Igfr1*), and follicular atresia (46 genes including *Bax/Bcl2* family members, caspases, and autophagy regulators). Pathway signatures were calculated by averaging normalized expression values (log2-CPM) of constituent genes within each pathway for each sample. Robustness of pathway scores was assessed by leave-one-out cross-validation to ensure stability against individual gene perturbations. For time course analysis, average pathway scores were calculated for each experimental group (MPF and GF) at each developmental time point (P7, P12, P21, P28, and P70), and then GF/MPF ratios were computed to identify time points with the most pronounced molecular divergence. To investigate the relationship between histological observations and gene signatures, we combined follicle compartmental modeling with gene expression profiling across multiple postnatal time points. Histological metrics (activation ratios, progression efficiency, and atresia rates) were aligned with molecular pathway signatures (activation genes, progression genes, and atresia genes) to identify concordance and temporal correlations. For integration, standardized z-scores were calculated for both histological efficiency ratios and gene expression pathway signatures, enabling direct comparison despite the different measurement scales. Pearson and Spearman correlations were calculated between standardized histological metrics and gene expression signatures across different developmental time points, with significance determined after multiple testing correction. To identify specific genes driving the activation-progression imbalance observed in GF mice, individual genes were scored based on: (1) correlation between gene expression pattern and histological metrics, (2) differential expression strength at critical developmental windows (P12 and P28), (3) membership in relevant biological pathways, and (4) magnitude of GF/MPF expression ratio changes over time.

#### Analysis of dietary effects on ovarian gene expression

To determine the contribution of manipulating diet composition at the beginning of the microbiota weaning transition on adult ovarian outcomes, we analyzed ovary samples from mice fed Chow, low-fat-low-fiber (Lf−Lfi), high-fat-low-fiber (Hf−Lfi), low-fat+fiber (Lf+Fi), or high-fat+fiber (Hf+Fi) diets. For these analyses, GF samples were excluded to focus specifically on diet effects within MPF mice. Principal component analysis was conducted to visualize clustering patterns among diet groups. Differential gene expression was performed as above, with the model design incorporating diet groups as factors. Pairwise comparisons were defined to assess: (1) effect of fiber in high-fat diet (Hf+Fi vs Hf-Lfi), (2) effect of fiber in low-fat diet (Lf+Fi vs Lf-Lfi), (3) effect of fat content with fiber (Hf+Fi vs Lf+Fi), (4) effect of fat content without fiber (Hf-Lfi vs Lf-Lfi), and (5) effect of each dietary condition versus Chow. Genes with FDR < 0.25 were considered differentially expressed. DEGs from each diet comparison were subjected to a comprehensive pathway analysis using clusterProfiler^148^ (version 3.18.1). GO term enrichment analysis was performed for Biological Process and Molecular Function terms, and KEGG pathway analysis identified overrepresented metabolic and signaling pathways. All analyses employed a significance threshold of p < 0.05, with the Benjamini-Hochberg correction for multiple testing (false discovery rate, FDR, < 0.25). For each significant GO term and KEGG pathway, enrichment metrics included gene ratio, fold enrichment, and z-scores to identify the most relevant pathways for the current study. Pathway analysis focused on functional categories involved in reproductive function within the GO and KEGG results, including mitochondrial function, oxidative stress response, energy metabolism, epigenetic regulation, oocyte quality, cell cycle, apoptosis/survival, and embryo development. A rescue analysis was conducted to identify pathways disrupted by a high-fat low-fiber diet (Hf-Lfi vs Chow) that were normalized by fiber supplementation (comparing Hf-Lfi vs. Hf+Fi and Hf+Fi vs. Chow). Pathways were classified as “rescued” when significantly different in both Hf-Lfi vs. Chow and Hf+Fi vs. Hf-Lfi comparisons, but not statistically different in Hf+Fi vs. Chow. Diet-dependent effects of fiber supplementation were investigated by comparing pathway enrichment patterns between high-fat and low-fat conditions, thereby demonstrating diet-specific responses to fiber in each dietary fat condition.

#### TaqMan assays of the arcuate nucleus of the hypothalamus

TaqMan gene expression analysis of the adult arcuate nucleus of the hypothalamus (Arc). Frozen brains from P70 female mice were cryosectioned at −20 °C. Using a hollow 1.0 mm needle, the Arc was removed following the stereotaxic coordinates in Paxinos’ mouse brain atlas.^150^ Arc micropunches were immediately dispensed into 500 μL of Trizol and stored at −80 °C until processing. Messenger RNA was purified using the RNeasy Mini kit (Qiagen). Standard manufacturer quantitative PCR protocols were used to analyze *Pdyn* (Mm00457573_m1) and *Kiss1* (Mm03058560_m1) using TaqMan probes (ThermoFisher).

### QUANTIFICATION AND STATISTICAL ANALYSIS

Statistical information, including sample size, mean, and statistical significance values, is shown in the text or the figure legends. A variety of statistical analyses were applied, each one appropriate for the data and hypothesis as depicted in corresponding figures, using R version 4.1.0 (R Core Team, 2021) or GraphPad Prism 9.3.1. For histological analyses and phenotypic measurements, statistical comparisons between groups were performed using t-test or one-way/two-way ANOVA with appropriate post-hoc tests. Time course analyses employed repeated measures ANOVA or mixed-effects models with multiple comparisons test. For RNA-seq analysis, differential gene expression was determined using limma-voom with empirical Bayes moderation of standard errors, controlling FDR at 0.25 for GF vs MPF as well as dietary comparisons. Significant pathways were identified using clusterProfiler with *p* < 0.05 and FDR < 0.25 after Benjamini-Hochberg correction. Correlation analyses between histological metrics and gene expression were performed using Pearson and Spearman methods with multiple testing correction. Alpha diversity analyses were conducted on CLR-normalized and rarefied data using statistical methods for nonparametric data, including the Kruskal-Wallis test with appropriate post-hoc testing. Beta diversity analyses were conducted using permutational multivariate analysis of variance (PERMANOVA) with 999 permutations. Differential abundance testing of microbial taxa was performed using ANOVA-Like Differential Expression tool for compositional data, version 1.28.0 (ALDEx2) with Welch’s t-test and Benjamini-Hochberg FDR correction (FDR < 0.25). This method employs a center log-ratio transformation and Monte Carlo sampling from a Dirichlet distribution to accurately account for the compositional nature of microbiome data. Pathway rescue analysis classified pathways as “rescued” based on statistical significance patterns across multiple comparisons. Quantification and statistical analyses of RNA-seq data were performed using R packages including tximport^143^ (transcript-level quantification), edgeR^144^ (filtering, normalization, count processing), limma (differential expression), and clusterProfiler^148,149^ (pathway analysis). Data visualization was done using ggplot2, pheatmap, and heatmaply.^147^ GraphPad Prism and Adobe Illustrator were used for generating figures.

## Supplementary Material

MMC1

MMC5

MMC4

MMC2

MMC3

Supplemental information can be found online at https://doi.org/10.1016/j.chom.2025.09.006.

## Figures and Tables

**Table:** KEY RESOURCES TABLE

REAGENT or RESOURCE	SOURCE	IDENTIFIER
Biological samples		
MPF/GF mouse stool		N/A
MPF/GF mouse brain tissue		N/A
MPF/GF mouse ovary		N/A
MPF/GF mouse plasma		N/A
MPF mouse testes/sperm		N/A
Chemicals, peptides, and recombinant proteins		
Chorionic gonadotropin human	Sigma-Aldrich	Cat# CG5
Pregnant mare serum gonadotropin	ProSpec-Tany	Cat# HOR-272
FERTIUP PM - CARD MEDIUM Set	Cosmo Bio USA	Ca# KYD-004-EX
M2 medium	Sigma-Aldrich	Cat# M7167
Hyaluronidase	Sigma-Aldrich	Cat# H4272
Sodium butyrate	Sigma-Aldrich	Cat# B5887
Sodium acetate	Sigma-Aldrich	Cat# P1880
Sodium propionate	Sigma-Aldrich	Cat# P1880
Rosshart Freezing Medium	https://doi.org/10.1038/s41467-025-60554-2	N/A
Critical commercial assays		
MagAttract PowerMicrobiome DNA/RNA EP Kit	Qiagen	Cat# 27500-4-EP
Qiagen RNeasy Mini Kit	Qiagen	Cat# 74106
TruSeq Stranded mRNA Kit	Illumina	Cat# 20020595
High Sensitivity RNA ScreenTape	Agilent	Cat# 5067-5579
RNA ScreenTape	Agilent	Cat# 5067-5576
RNA ScreenTape Ladder	Agilent	Cat# 5067-5578
RNA ScreenTape Sample Buffer	Agilent	Cat# 5067-5577
D5000 ScreenTape	Agilent	Cat# 5067-5588
D5000 Reagents	Agilent	Cat# 5067-5589
Qubit 1X dsDNA BR Assay Kit	ThermoFisher	Cat# Q33266
Qubit RNA BR Assay Kit	ThermoFisher	Cat# Q10211
AMPure XP reagent	Beckman Coulter	Cat# A63881
Illumina Stranded mRNA Prep, Ligation kit	Illumina	Cat# 20040534
IDT for Illumina RNA UD Indexes Set A	Illumina	Cat # 20040553
Nextera XT DNA Library Preparation Kit	llumina	Cat# FC-131-1096
Nextera XT Index Kit v2 Set A	llumina	Cat# FC-131-2001
KAPA HiFi HotStart ReadyMix PCR Kit	Roche Diagnostics	Cat# 9420398001
Illumina DNA/RNA UD Indexes Set A	Illumina	Cat# # 20091654
Masson’s Trichrome Stain Kit	Polysciences	Cat# 25088
Deposited data		
mRNAseq FASTQ files	This paper	Accession: PRJNA1310612
16S rRNA FASTQ files	This paper	Accession: PRJNA1309685
Experimental models: Organisms/strains		
Mouse: C57BL/6NTac MPF	Taconic	Stock No: B6-MPF
Mouse: C57BL/6NTac GF	Taconic	Stock No: B6-GF
Oligonucleotides		
16S Amplicon PCR Forward Primer: 5′ TCGTCGGCAGCGTCAGATGTGTA TAAGAGACAGCCTACGGGNGGC WGCAG; 16S Amplicon PCR Reverse Primer: 5′ GTCTCGTGGGCTCGGAGA TGTGTATAAGAGACAGGACTACHVG GGTATCTAATCC	Integrated DNA Technologies	N/A
Kiss1	Thermo Scientific	Mm03058560_m1
Pdyn	Thermo Scientific	Mm00457573_m1
Software and algorithms		
bcl2fastq2 conversion software v2.2.0		RRID:SCR_015058
QIIME 2 v2022.2		RRID:SCR_021258
R v4.2.1		RRID:SCR_001905
RStudio v1.2.5001		RRID:SCR_000432
tximport v3.4		RRID:SCR_016752
kallisto v0.46.1		RRID:SCR_016582
phyloseq v1.40.0		RRID:SCR_013080
Decontam v1.16.0		https://bioconductor.org/packages/decontam
SILVA reference database v13.8		RRID:SCR_006423
ALDEx2 v1.28.0		RRID:SCR_003364
ImageJ v2.14		RRID:SCR_003070
FastQC v0.11.9		RRID:SCR_014583
MultiQC v1.11		RRID:SCR_014982
edgeR v3.34.1		RRID:SCR_012802
limma v3.48.3		RRID:SCR_010943
clusterProfiler v3.18.1		RRID:SCR_016884
GraphPad Prism v10.4.1		RRID:SCR_002798
Python v2.7		RRID:SCR_008394
MAFFT v7		RRID:SCR_011811
FastTree: 2		RRID:SCR_015501
DADA2 v1.36.0		RRID:SCR_023519
MicrobiomeAnalyst v2.0		RRID:SCR_015022
GOmixer v1.7.5.0		https://www.raeslab.org/gomixer/
PANTHER DB v19.0		RRID:SCR_004869
PICRUSt2 v2.6.2		RRID:SCR_022647
q2-feature-classifier v2024.10.1		https://docs.qiime2.org/2024.10/plugins/available/feature-classifier/
Bowtie 2 v2.5.4		RRID:SCR_016368
SAMtools v1.22.1		RRID:SCR_002105
Trimmomatic v 0.39		RRID:SCR_011848
Kraken v2: 2.1.6		RRID:SCR_026838
Prodigal v2.6.3		RRID:SCR_011936
WGSA2 pipeline v3.0.3		https://nephele.niaid.nih.gov/user-guide/pipeline-descriptions/wgsa2
Heatmaply v1.6.0		https://cran.r-project.org/web/packages/heatmaply/index.html
voom v3.64.1		RRID:SCR_010943 (function in limma)
tidyverse v2.0.0		RRID:SCR_019186
minpack.lm v1.2-4		https://cran.r-project.org/package=minpack.lm
patchwork v1.3.1		RRID:SCR_000072
Nephele platform v 3.0.3		RRID:SCR_016595
Other		
NIH-31 Irradiated modified Rodent Diet	Teklad Envigo	Cat. #7913
low-fat low-fiber diet (Lf-Lfi	Teklad Envigo	Cat. #TD.240245
low-fat with fiber diet (Lf+Fi)	Teklad Envigo	Cat. #TD.240246
high-fat low-fiber diet (Hf-Lfi)	Teklad Envigo	Cat. #TD240247

## Data Availability

The mRNA-seq, 16S gene marker profiling, and shotgun metagenomics data generated in this study were deposited in the NCBI SRA database under accession PRJNA1310612 and PRJNA1309685, respectively. This paper does not report any original code. Any requests for data will be fulfilled by the lead contact upon request.

## Highlights

- Lifetime reproductive success of mice depends on the microbiota
- Colonization during the weaning transition prevents premature ovarian follicle loss
- Microbial metabolites, notably SCFAs, prevent accelerated loss of the ovarian reserve
- Dietary fiber sustains oocyte quality in high-fat diet conditions

## References

[1] World Health Organization (1990–2021). Infertility Prevalence Estimates. https://www.who.int/publications/i/item/978920068315.

[2] Guillette LJ, and Edwards TM (2008). Environmental influences on fertility: can we learn lessons from studies of wildlife? Fertil. Steril 89, e21–e24. 10.1016/j.fertnstert.2007.12.019. 18308053

[3] Belkaid Y, and Hand TW (2014). Role of the Microbiota in Immunity and Inflammation. Cell 157, 121–141. 10.1016/j.cell.2014.03.011. 24679531 PMC4056765

[4] Collins N, and Belkaid Y (2022). Control of immunity via nutritional interventions. Immunity 55, 210–223. 10.1016/j.immuni.2022.01.004. 35139351

[5] Round JL, and Mazmanian SK (2009). The gut microbiota shapes intestinal immune responses during health and disease. Nat. Rev. Immunol 9, 313–323. 10.1038/nri2515. 19343057 PMC4095778

[6] Hooper LV, Midtvedt T, and Gordon JI (2002). HOW host-microbial interactions shape the nutrient environment of the mammalian intestine. Annu. Rev. Nutr 22, 283–307. 10.1146/annurev.nutr.22.011602.092259. 12055347

[7] Jašarević E, Howard CD, Misic AM, Beiting DP, and Bale TL (2017). Stress during pregnancy alters temporal and spatial dynamics of the maternal and offspring microbiome in a sex-specific manner. Sci. Rep 7, 44182. 10.1038/srep44182. 28266645 PMC5339804

[8] Jašarević E, Hill EM, Kane PJ, Rutt L, Gyles T, Folts L, Rock KD, Howard CD, Morrison KE, Ravel J, (2021). The composition of human vaginal microbiota transferred at birth affects offspring health in a mouse model. Nat. Commun 12, 6289. 10.1038/s41467-021-26634-9. 34725359 PMC8560944

[9] Pronovost GN, Yu KB, Coley-O’Rourke EJL, Telang SS, Chen AS, Vuong HE, Williams DW, Chandra A, Rendon TK, Paramo J, (2023). The maternal microbiome promotes placental development in mice. Sci. Adv 9, eadk1887. 10.1126/sciadv.adk1887. 37801498 PMC10558122

[10] Lopez-Tello J, Schofield Z, Kiu R, Dalby MJ, van Sinderen D, Le Gall G, Sferruzzi-Perri AN, and Hall LJ (2022). Maternal gut microbiota Bifidobacterium promotes placental morphogenesis, nutrient transport and fetal growth in mice. Cell. Mol. Life Sci 79, 386. 10.1007/s00018-022-04379-y. 35760917 PMC9236968

[11] Lim AI, McFadden T, Link VM, Han S-J, Karlsson R-M, Stacy A, Farley TK, Lima-Junior DS, Harrison OJ, Desai JV, (2021). Prenatal maternal infection promotes tissue-specific immunity and inflammation in offspring. Science 373, eabf3002. 10.1126/science.abf3002. 34446580

[12] Koren O, Goodrich JK, Cullender TC, Spor A, Laitinen K, Bäckhed HK, Gonzalez A, Werner JJ, Angenent LT, Knight R, (2012). Host remodeling of the gut microbiome and metabolic changes during pregnancy. Cell 150, 470–480. 10.1016/j.cell.2012.07.008. 22863002 PMC3505857

[13] Vuong HE, Pronovost GN, Williams DW, Coley EJL, Siegler EL, Qiu A, Kazantsev M, Wilson CJ, Rendon T, and Hsiao EY (2020). The maternal microbiome modulates fetal neurodevelopment in mice. Nature 586, 281–286. 10.1038/s41586-020-2745-3. 32968276 PMC7554197

[14] Zou J, Ngo VL, Wang Y, Wang Y, and Gewirtz AT (2023). Maternal fiber deprivation alters microbiota in offspring, resulting in low-grade inflammation and predisposition to obesity. Cell Host Microbe 31, 45–57.e7. 10.1016/j.chom.2022.10.014. 36493784 PMC9850817

[15] Jašarević E, Howard CD, Morrison K, Misic A, Weinkopff T, Scott P, Hunter C, Beiting D, and Bale TL (2018). The maternal vaginal microbiome partially mediates the effects of prenatal stress on offspring gut and hypothalamus. Nat. Neurosci 21, 1061–1071. 10.1038/s41593-018-0182-5. 29988069

[16] Metcalf CJE, Henry LP, Rebolleda-Gómez M, and Koskella B (2019). Why Evolve Reliance on the Microbiome for Timing of Ontogeny? mBio 10, e01496–e01419. 10.1128/mBio.01496-19. 31594812 PMC6786867

[17] Shin SC, Kim S-H, You H, Kim B, Kim AC, Lee K-A, Yoon J-H, Ryu J-H, and Lee W-J (2011). Drosophila microbiome modulates host developmental and metabolic homeostasis via insulin signaling. Science 334, 670–674. 10.1126/science.1212782. 22053049

[18] Brown MR, Clark KD, Gulia M, Zhao Z, Garczynski SF, Crim JW, Suderman RJ, and Strand MR (2008). An insulin-like peptide regulates egg maturation and metabolism in the mosquito Aedes aegypti. Proc. Natl. Acad. Sci. USA 105, 5716–5721. 10.1073/pnas.0800478105. 18391205 PMC2311378

[19] Dedeine F, Vavre F, Fleury F, Loppin B, Hochberg ME, and Bouletreau M (2001). Removing symbiotic Wolbachia bacteria specifically inhibits oogenesis in a parasitic wasp. Proc. Natl. Acad. Sci. USA 98, 6247–6252. 10.1073/pnas.101304298. 11353833 PMC33453

[20] Gnainsky Y, Zfanya N, Elgart M, Omri E, Brandis A, Mehlman T, Itkin M, Malitsky S, Adamski J, and Soen Y (2021). Systemic Regulation of Host Energy and Oogenesis by Microbiome-Derived Mitochondrial Coenzymes. Cell Rep. 34, 108583. 10.1016/j.celrep.2020.108583. 33406416

[21] Elgart M, Stern S, Salton O, Gnainsky Y, Heifetz Y, and Soen Y (2016). Impact of gut microbiota on the fly’s germ line. Nat. Commun 7, 11280. 10.1038/ncomms11280. 27080728 PMC4835552

[22] Li W-L, Huang Q, Li J-L, Wu P, Wei B, Li X-J, Tang Q-H, Dong Z-X, Xiong J, Tang H, (2023). Gut microbiota-driven regulation of queen bee ovarian metabolism. Microbiol. Spectr 11, e0214523. 10.1128/spectrum.02145-23. 37750696 PMC10581225

[23] Lopez-Tello J, Kiu R, Schofield Z, Zhang CXW, van Sinderen D, Le Gall G, Hall LJ, and Sferruzzi-Perri AN (2024). Maternal gut Bifidobacterium breve modifies fetal brain metabolism in germ-free mice. Mol. Metab 88, 102004. 10.1016/j.molmet.2024.102004. 39127167 PMC11401360

[24] Feng Y, Zheng H, Yin C, Liang D, Zhang S, Chen J, Mai F, Lan Z, Zhu M, Mai Z, (2024). β-resorcylic acid released by Limosilactobacillus reuteri protects against cisplatin-induced ovarian toxicity and infertility. Cell Rep. Med. 5, 101678. 10.1016/j.xcrm.2024.101678. 39096912 PMC11384965

[25] Argaw-Denboba A, Schmidt TSB, Di Giacomo M, Ranjan B, Devendran S, Mastrorilli E, Lloyd CT, Pugliese D, Paribeni V, Dabin J, (2024). Paternal microbiome perturbations impact offspring fitness. Nature 629, 652–659. 10.1038/s41586-024-07336-w. 38693261 PMC11096121

[26] Gambineri A, Laudisio D, Marocco C, Radellini S, Colao A, and Savastano S; Obesity Programs of nutrition, Education, Research and Assessment (OPERA) group (2019). Female infertility: which role for obesity? Int. J. Obes. Suppl 9, 65–72. 10.1038/s41367-019-0009-1. 31391925 PMC6683114

[27] Nteeba J, Ganesan S, and Keating AF (2014). Progressive obesity alters ovarian folliculogenesis with impacts on pro-inflammatory and steroidogenic signaling in female mice. Biol. Reprod 91, 86. 10.1095/biolreprod.114.121343. 25143355 PMC4435031

[28] Fan Y, and Pedersen O (2021). Gut microbiota in human metabolic health and disease. Nat. Rev. Microbiol 19, 55–71. 10.1038/s41579-020-0433-9. 32887946

[29] Pasquali R, Pelusi C, Genghini S, Cacciari M, and Gambineri A (2003). Obesity and reproductive disorders in women. Hum. Reprod. Update 9, 359–372. 10.1093/humupd/dmg024. 12926529

[30] Maruvada P, Leone V, Kaplan LM, and Chang EB (2017). The Human Microbiome and Obesity: Moving beyond Associations. Cell Host Microbe 22, 589–599. 10.1016/j.chom.2017.10.005. 29120742

[31] Segal TR, and Giudice LC (2022). Systematic review of climate change effects on reproductive health. Fertil. Steril 118, 215–223. 10.1016/j.fertnstert.2022.06.005. 35878942

[32] Eppig JJ, Wigglesworth K, and Pendola FL (2002). The mammalian oocyte orchestrates the rate of ovarian follicular development. Proc. Natl. Acad. Sci. USA 99, 2890–2894. 10.1073/pnas.052658699. 11867735 PMC122443

[33] Edson MA, Nagaraja AK, and Matzuk MM (2009). The Mammalian Ovary from Genesis to Revelation. Endocr. Rev 30, 624–712. 10.1210/er.2009-0012. 19776209 PMC2761115

[34] Shimizu K, Muranaka Y, Fujimura R, Ishida H, Tazume S, and Shimamura T (1998). Normalization of reproductive function in germfree mice following bacterial contamination. Exp. Anim 47, 151–158. 10.1538/expanim.47.151. 9816490

[35] Shinoda M, Tamura H, Maejima K, and Watarai S (1980). Reproductive ability of germfree ICR female mice (author’s transl). Jikken Dobutsu 29, 55–59. 10.1538/expanim1978.29.1_55. 7053212

[36] Al-Asmakh M, Stukenborg J-B, Reda A, Anuar F, Strand M-L, Hedin L, Pettersson S, and Soder O (2014). The Gut Microbiota and Developmental Programming of the Testis in Mice. PLoS One 9, e103809. 10.1371/journal.pone.0103809. 25118984 PMC4132106

[37] Trigg NA, Zhou SK, Harris JC, Lamonica MN, Nelson MA, Silverman MA, Kambayashi T, and Conine CC (2025). A lack of commensal microbiota influences the male reproductive tract intergenerationally in mice. Reproduction 169, e240204. 10.1530/REP-24-0204. 39946159 PMC11906130

[38] Bristol-Gould SK, Kreeger PK, Selkirk CG, Kilen SM, Cook RW, Kipp JL, Shea LD, Mayo KE, and Woodruff TK (2006). Postnatal regulation of germ cells by activin: the establishment of the initial follicle pool. Dev. Biol 298, 132–148. 10.1016/j.ydbio.2006.06.025. 16930587

[39] Bristol-Gould SK, Kreeger PK, Selkirk CG, Kilen SM, Mayo KE, Shea LD, and Woodruff TK (2006). Fate of the initial follicle pool: empirical and mathematical evidence supporting its sufficiency for adult fertility. Dev. Biol 298, 149–154. 10.1016/j.ydbio.2006.06.023. 16925987

[40] Zeleznik AJ (2004). The physiology of follicle selection. Reprod. Biol. Endocrinol 2, 31. 10.1186/1477-7827-2-31. 15200680 PMC442133

[41] Zeleznik AJ (2001). Follicle selection in primates: “many are called but few are chosen.”. Biol. Reprod 65, 655–659. 10.1095/biolreprod65.3.655. 11514325

[42] Rajkovic A, Pangas SA, Ballow D, Suzumori N, and Matzuk MM (2004). NOBOX deficiency disrupts early folliculogenesis and oocyte-specific gene expression. Science 305, 1157–1159. 10.1126/science.1099755. 15326356

[43] McIntosh CJ, Lun S, Lawrence S, Western AH, McNatty KP, and Juengel JL (2008). The Proregion of Mouse BMP15 Regulates the Cooperative Interactions of BMP15 and GDF91. Biol. Reprod 79, 889–896. 10.1095/biolreprod.108.068163. 18633140

[44] McMahon HE, Hashimoto O, Mellon PL, and Shimasaki S (2008). Oocyte-Specific Overexpression of Mouse Bone Morphogenetic Protein-15 Leads to Accelerated Folliculogenesis and an Early Onset of Acyclicity in Transgenic Mice. Endocrinology 149, 2807–2815. 10.1210/en.2007-1550. 18308851 PMC2408818

[45] John GB, Shidler MJ, Besmer P, and Castrillon DH (2009). Kit signaling via PI3K promotes ovarian follicle maturation but is dispensable for primordial follicle activation. Dev. Biol 331, 292–299. 10.1016/j.ydbio.2009.05.546. 19447101 PMC2726617

[46] Durlinger ALL, Kramer P, Karels B, de Jong FH, Uilenbroek J.Th.J., Grootegoed JA, and Themmen APN (1999). Control of Primordial Follicle Recruitment by Anti-Mü llerian Hormone in the Mouse Ovary1. Endocrinology 140, 5789–5796. 10.1210/endo.140.12.7204. 10579345

[47] Ricard-Blum S, Baffet G, and Théret N (2018). Molecular and tissue alterations of collagens in fibrosis. Matrix Biol. 68–69, 122–149. 10.1016/j.matbio.2018.02.004. 29458139

[48] Plant TM (2015). 60 YEARS OF NEUROENDOCRINOLOGY: The hypothalamo-pituitary–gonadal axis. J. Endocrinol. 226, T41–T54. 10.1530/JOE-15-0113. 25901041 PMC4498991

[49] Navarro VM (2012). New Insights into the Control of Pulsatile GnRH Release: The Role of Kiss1/Neurokinin B Neurons. Front. Endocrinol 3, 48. 10.3389/fendo.2012.00048. PMC3355984 22649420

[50] Skorupskaite K, George JT, and Anderson RA (2014). The kisspeptin-GnRH pathway in human reproductive health and disease. Hum. Reprod. Update 20, 485–500. 10.1093/humupd/dmu009. 24615662 PMC4063702

[51] Kennedy GC, and Mitra J (1963). Body weight and food intake as initiating factors for puberty in the rat. J. Physiol 166, 408–418. 10.1113/jphysiol.1963.sp007112. 14031944 PMC1359337

[52] Dicken C, Menke M, and Neal-Perry G (2010). The Hypothalamic-Pituitary-Ovarian Axis. In Amenorrhea, Clinical Guide, Santoro NF, ed. (Humana Press), pp. 1–19. 10.1007/978-1-60327-864-5_1.

[53] Fortune JE (1994). Ovarian Follicular Growth and Development in Mammals1. Biol. Reprod 50, 225–232. 10.1095/bio-lreprod50.2.225. 8142540

[54] Myers M, Britt KL, Wreford NGM, Ebling FJP, and Kerr JB (2004). Methods for quantifying follicular numbers within the mouse ovary. Reproduction 127, 569–580. 10.1530/rep.1.00095. 15129012

[55] Hirshfield AN, and Midgley AR Jr. (1978). Morphometric Analysis of Foilicular Development in the Rat. Biol. Reprod 19, 597–605. 10.1095/biolreprod19.3.597. 363183

[56] Abercrombie M (1946). Estimation of nuclear population from microtome sections. Anat. Rec 94, 239–247. 10.1002/ar.1090940210. 21015608

[57] Wear HM, McPike MJ, and Watanabe KH (2016). From primordial germ cells to primordial follicles: a review and visual representation of early ovarian development in mice. J. Ovarian Res 9, 36. 10.1186/s13048-016-0246-7. 27329176 PMC4915180

[58] Boateng R, Boechat N, Henrich PP, and Bolcun-Filas E (2021). Whole Ovary Immunofluorescence, Clearing, and Multiphoton Microscopy for Quantitative 3D Analysis of the Developing Ovarian Reserve in Mouse. J. Vis. Exp 10.3791/62972. PMC8911993 34542534

[59] Chen Y, Liu Q, Liu R, Yang C, Wang X, Ran Z, Zhou S, Li X, and He C (2021). A Prepubertal Mice Model to Study the Growth Pattern of Early Ovarian Follicles. Int. J. Mol. Sci 22, 5130. 10.3390/ijms22105130. 34066233 PMC8151218

[60] Faddy MJ, and Gosden RG (1996). A model conforming the decline in follicle numbers to the age of menopause in women. Hum. Reprod 11, 1484–1486. 10.1093/oxfordjournals.humrep.a019422. 8671489

[61] Hansen KR, Knowlton NS, Thyer AC, Charleston JS, Soules MR, and Klein NA (2008). A new model of reproductive aging: the decline in ovarian non-growing follicle number from birth to menopause. Hum. Reprod 23, 699–708. 10.1093/humrep/dem408. 18192670

[62] Knowlton NS, Craig LB, Zavy MT, and Hansen KR (2014). Validation of the power model of ovarian nongrowing follicle depletion associated with aging in women. Fertil. Steril 101, 851–856. 10.1016/j.fertnstert.2013.12.008. 24424370

[63] Lawley SD, Sammel MD, Santoro N, and Johnson J (2024). Mathematical recapitulation of the end stages of human ovarian aging. Sci. Adv 10, eadj4490. 10.1126/sciadv.adj4490. 38215196 PMC10786411

[64] Ballif G, Clément F, and Yvinec R (2024). Nonlinear compartmental modeling to monitor ovarian follicle population dynamics on the whole lifespan. J. Math. Biol 89, 9. 10.1007/s00285-024-02108-6. 38844702

[65] Fernandes AD, Reid JN, Macklaim JM, McMurrough TA, Edgell DR, and Gloor GB (2014). Unifying the analysis of high-throughput sequencing datasets: characterizing RNA-seq, 16S rRNA gene sequencing and selective growth experiments by compositional data analysis. Microbiome 2, 15. 10.1186/2049-2618-2-15. 24910773 PMC4030730

[66] Al Nabhani Z, Dulauroy S, Marques R, Cousu C, Al Bounny S, Déjardin F, Sparwasser T, Bérard M, Cerf-Bensussan N, and Eberl G (2019). A Weaning Reaction to Microbiota Is Required for Resistance to Immunopathologies in the Adult. Immunity 50, 1276–1288.e5. 10.1016/j.immuni.2019.02.014. 30902637

[67] Flores JN, Lubin J-B, and Silverman MA (2024). The case for microbial intervention at weaning. Gut Microbes 16, 2414798. 10.1080/19490976.2024.2414798. 39468827 PMC11540084

[68] Knoop KA, Gustafsson JK, McDonald KG, Kulkarni DH, Coughlin PE, McCrate S, Kim D, Hsieh C-S, Hogan SP, Elson CO, (2017). Microbial antigen encounter during a preweaning interval is critical for tolerance to gut bacteria. Sci. Immunol 2, eaao1314. 10.1126/sciimmunol.aao1314. 29246946 PMC5759965

[69] Pantoja-Feliciano IG, Clemente JC, Costello EK, Perez ME, Blaser MJ, Knight R, and Dominguez-Bello MG (2013). Biphasic assembly of the murine intestinal microbiota during early development. ISME J. 7, 1112–1115. 10.1038/ismej.2013.15. 23535917 PMC3660675

[70] Gensollen T, Iyer SS, Kasper DL, and Blumberg RS (2016). How colonization by microbiota in early life shapes the immune system. Science 352, 539–544. 10.1126/science.aad9378. 27126036 PMC5050524

[71] McDonald B, and McCoy KD (2019). Maternal microbiota in pregnancy and early life. Science 365, 984–985. 10.1126/sci-ence.aay0618. 31488675

[72] Henning SJ (1981). Postnatal development: coordination of feeding, digestion, and metabolism. Am. J. Physiol 241, G199–G214. 10.1152/ajpgi.1981.241.3.G199. 7025659

[73] Galef BG (1979). Investigation of the functions of coprophagy in juvenile rats. J. Comp. Physiol. Psychol 93, 295–305. 10.1037/h0077551.

[74] Babický A, Parizek J, Ostádalová I, and Kolár J (1973). Initial solid food intake and growth of young rats in nests of different sizes. Physiol. Bohemoslov 22, 557–566. 4273064

[75] Leon M (1974). Maternal pheromone. Physiol. Behav 13, 441–453. 10.1016/0031-9384(74)90098-5. 4474674

[76] Hall WG (1975). Weaning and Growth of Artificially Rreared Rats. Science 190, 1313–1315. 10.1126/science.1198116. 1198116

[77] Hoces D, Lan J, Sun W, Geiser T, Stä ubli ML, Cappio Barazzone EC, Arnoldini M, Challa TD, Klug M, Kellenberger A, (2022). Metabolic reconstitution of germ-free mice by a gnotobiotic microbiota varies over the circadian cycle. PLoS Biol. 20, e3001743. 10.1371/journal.pbio.3001743. 36126044 PMC9488797

[78] Hayes E, Kushnir V, Ma X, Biswas A, Prizant H, Gleicher N, and Sen A (2016). Intra-cellular mechanism of Anti-Mü llerian hormone (AMH) in regulation of follicular development. Mol. Cell. Endocrinol 433, 56–65. 10.1016/j.mce.2016.05.019. 27235859

[79] Guo R, and Pankhurst MW (2020). Accelerated ovarian reserve depletion in female anti-Mü llerian hormone knockout mice has no effect on lifetime fertility†. Biol. Reprod 102, 915–922. 10.1093/biolre/ioz227. 31837140

[80] Jones RL, and Pepling ME (2013). KIT signaling regulates primordial follicle formation in the neonatal mouse ovary. Dev. Biol 382, 186–197. 10.1016/j.ydbio.2013.06.030. 23831378

[81] Charbonneau MR, Blanton LV, DiGiulio DB, Relman DA, Lebrilla CB, Mills DA, and Gordon JI (2016). A microbial perspective of human developmental biology. Nature 535, 48–55. 10.1038/nature18845. 27383979 PMC5358965

[82] Narushima S, Sugiura Y, Oshima K, Atarashi K, Hattori M, Suematsu M, and Honda K (2014). Characterization of the 17 strains of regulatory T cell-inducing human-derived Clostridia. Gut Microbes 5, 333–339. 10.4161/gmic.28572. 24642476 PMC4153770

[83] van Munster IP, Tangerman A, and Nagengast FM (1994). Effect of resistant starch on colonic fermentation, bile acid metabolism, and mucosal proliferation. Dig. Dis. Sci 39, 834–842. 10.1007/BF02087431. 8149850

[84] Caderni G, Luceri C, Lancioni L, and Dolara P (1996). Dietary sucrose, glucose, fructose, and starches affect colonic functions in rats. Nutr. Cancer 25, 179–186. 10.1080/01635589609514440. 8710687

[85] McClave SA, Greene LM, Snider HL, Makk LJ, Cheadle WG, Owens NA, Dukes LG, and Goldsmith LJ (1997). Comparison of the safety of early enteral vs parenteral nutrition in mild acute pancreatitis. JPEN J. Parenter. Enter. Nutr 21, 14–20. 10.1177/014860719702100114. 9002079

[86] Vonk RJ, Hagedoorn RE, de Graaff R, Elzinga H, Tabak S, Yang YX, and Stellaard F (2000). Digestion of so-called resistant starch sources in the human small intestine. Am. J. Clin. Nutr 72, 432–438. 10.1093/ajcn/72.2.432. 10919938

[87] Le Leu RK, Hu Y, Brown IL, and Young GP (2009). Effect of high amylose maize starches on colonic fermentation and apoptotic response to DNA-damage in the colon of rats. Nutr. Metab. (Lond) 6, 11. 10.1186/1743-7075-6-11. 19267935 PMC2656505

[88] Sobh M, Montroy J, Daham Z, Sibbald S, Lalu M, Stintzi A, Mack D, and Fergusson DA (2022). Tolerability and SCFA production after resistant starch supplementation in humans: a systematic review of randomized controlled studies. Am. J. Clin. Nutr 115, 608–618. 10.1093/ajcn/nqab402. 34871343

[89] Malcomson FC, Willis ND, McCallum I, Xie L, Ouwehand AC, Stowell JD, Kelly S, Bradburn DM, Belshaw NJ, Johnson IT, (2020). Resistant starch supplementation increases crypt cell proliferative state in the rectal mucosa of older healthy participants. Br. J. Nutr 124, 374–385. 10.1017/S0007114520001312. 32279690 PMC7369377

[90] Li F, Armet AM, Korpela K, Liu J, Quevedo RM, Asnicar F, Seethaler B, Rusnak TBS, Cole JL, Zhang Z, (2025). Cardiometabolic benefits of a non-industrialized-type diet are linked to gut microbiome modulation. Cell 188, 1226–1247.e18. 10.1016/j.cell.2024.12.034. 39855197

[91] Oliphant K, and Allen-Vercoe E (2019). Macronutrient metabolism by the human gut microbiome: major fermentation by-products and their impact on host health. Microbiome 7, 91. 10.1186/s40168-019-0704-8. 31196177 PMC6567490

[92] Dalby MJ, Ross AW, Walker AW, and Morgan PJ (2017). Dietary Uncoupling of Gut Microbiota and Energy Harvesting from Obesity and Glucose Tolerance in Mice. Cell Rep. 21, 1521–1533. 10.1016/j.celrep.2017.10.056. 29117558 PMC5695904

[93] Morrison KE, Jašarević E, Howard CD, and Bale TL (2020). It’s the fiber, not the fat: significant effects of dietary challenge on the gut microbiome. Microbiome 8, 15. 10.1186/s40168-020-0791-6. 32046785 PMC7014620

[94] Snider AP, and Wood JR (2019). Obesity induces ovarian inflammation and reduces oocyte quality. Reproduction 158, R79–R90. 10.1530/REP-18-0583. 30999278

[95] Wang L, Tang J, Wang L, Tan F, Song H, Zhou J, and Li F (2021). Oxidative stress in oocyte aging and female reproduction. J. Cell. Physiol 236, 7966–7983. 10.1002/jcp.30468. 34121193

[96] Nteeba J, Ortinau LC, Perfield JW, and Keating AF (2013). Diet-induced obesity alters immune cell infiltration and expression of inflammatory cytokine genes in mouse ovarian and peri-ovarian adipose depot tissues. Mol. Reprod. Dev 80, 948–958. 10.1002/mrd.22231. 24038509

[97] Xie F, Anderson CL, Timme KR, Kurz SG, Fernando SC, and Wood JR (2016). Obesity-Dependent Increases in Oocyte mRNAs Are Associated With Increases in Proinflammatory Signaling and Gut Microbial Abundance of Lachnospiraceae in Female Mice. Endocrinology 157, 1630–1643. 10.1210/en.2015-1851. 26881311 PMC4816731

[98] Meulders B, Marei WFA, Loier L, and Leroy JLMR (2025). Lipotoxicity and Oocyte Quality in Mammals: Pathogenesis, Consequences, and Reversibility. Annu. Rev. Anim. Biosci 13, 233–254. 10.1146/annurev-animal-111523-102249. 39565833

[99] Luke B, Brown MB, Wantman E, Lederman A, Gibbons W, Schattman GL, Lobo RA, Leach RE, and Stern JE (2012). Cumulative Birth Rates with Linked Assisted Reproductive Technology Cycles. N. Engl. J. Med 366, 2483–2491. 10.1056/NEJMoa1110238. 22738098 PMC3623697

[100] Rittenberg V, Seshadri S, Sunkara SK, Sobaleva S, Oteng-Ntim E, and El-Toukhy T (2011). Effect of body mass index on IVF treatment outcome: an updated systematic review and meta-analysis. Reprod. Biomed. Online 23, 421–439. 10.1016/j.rbmo.2011.06.018. 21885344

[101] Provost MP, Acharya KS, Acharya CR, Yeh JS, Steward RG, Eaton JL, Goldfarb JM, and Muasher SJ (2016). Pregnancy outcomes decline with increasing body mass index: analysis of 239,127 fresh autologous in vitro fertilization cycles from the 2008–2010 Society for Assisted Reproductive Technology registry. Fertil. Steril 105, 663–669. 10.1016/j.fertnstert.2015.11.008. 26627120

[102] Bellver J, Ayllón Y, Ferrando M, Melo M, Goyri E, Pellicer A, Remohi J, and Meseguer M (2010). Female obesity impairs in vitro fertilization outcome without affecting embryo quality. Fertil. Steril 93, 447–454. 10.1016/j.fertnstert.2008.12.032. 19171335

[103] Shah DK, Missmer SA, Berry KF, Racowsky C, and Ginsburg ES (2011). Effect of obesity on oocyte and embryo quality in women undergoing in vitro fertilization. Obstet. Gynecol 118, 63–70. 10.1097/AOG.0b013e31821fd360. 21691164

[104] Leary C, Leese HJ, and Sturmey RG (2015). Human embryos from overweight and obese women display phenotypic and metabolic abnormalities. Hum. Reprod 30, 122–132. 10.1093/humrep/deu276. 25391239

[105] Robker RL (2008). Evidence that obesity alters the quality of oocytes and embryos. Pathophysiology 15, 115–121. 10.1016/j.pathophys.2008.04.004. 18599275

[106] Gonzalez MB, Robker RL, and Rose RD (2022). Obesity and oocyte quality: significant implications for ART and emerging mechanistic insights. Biol Reprod. 106, 338–350. 10.1093/biolre/ioab228. 34918035

[107] Ahmed B, and Konje JC (2023). The epidemiology of obesity in reproduction. Best Pract. Res. Clin. Obstet. Gynaecol 89, 102342. 10.1016/j.bpobgyn.2023.102342. 37276817

[108] Hesketh KD, Zheng M, and Campbell KJ (2025). Early life factors that affect obesity and the need for complex solutions. Nat. Rev. Endocrinol 21, 31–44. 10.1038/s41574-024-01035-2. 39313572

[109] Dağ ZÖ, and Dilbaz B (2015). Impact of obesity on infertility in women. J. Turk. Ger. Gynecol. Assoc 16, 111–117. 10.5152/jtgga.2015.15232. 26097395 PMC4456969

[110] Kukaev E, Kirillova E, Tokareva A, Rimskaya E, Starodubtseva N, Chernukha G, Priputnevich T, Frankevich V, and Sukhikh G (2024). Impact of Gut Microbiota and SCFAs in the Pathogenesis of PCOS and the Effect of Metformin Therapy. Int. J. Mol. Sci 25, 10636. 10.3390/ijms251910636. 39408965 PMC11477200

[111] Olaniyi KS, Bashir AM, Areloegbe SE, Sabinari IW, Akintayo CO, Oniyide AA, and Aturamu A (2022). Short chain fatty acid, acetate restores ovarian function in experimentally induced PCOS rat model. PLoS One 17, e0272124. 10.1371/journal.pone.0272124. 35881588 PMC9321379

[112] Wu J, Zhuo Y, Liu Y, Chen Y, Ning Y, and Yao J (2021). Association between premature ovarian insufficiency and gut microbiota. BMC Pregnancy Childbirth 21, 418. 10.1186/s12884-021-03855-w. 34090383 PMC8180047

[113] Wang J, Luo R, Zhao X, Xia D, Liu Y, Shen T, and Liang Y (2023). Association between gut microbiota and primary ovarian insufficiency: a bidirectional two-sample Mendelian randomization study. Front. Endocrinol 14, 1183219. 10.3389/fendo.2023.1183219. PMC10324962 37424857

[114] Rosshart SP, Herz J, Vassallo BG, Hunter A, Wall MK, Badger JH, McCulloch JA, Anastasakis DG, Sarshad AA, Leonardi I, (2019). Laboratory mice born to wild mice have natural microbiota and model human immune responses. Science 365, eaaw4361. 10.1126/science.aaw4361. 31371577 PMC7377314

[115] Runge S, von Zedtwitz S, Maucher AM, Bruno P, Osbelt L, Zhao B, Gernand AM, Lesker TR, Gräwe K, Rogg M, (2025). Laboratory mice engrafted with natural gut microbiota possess a wildling-like phenotype. Nat. Commun 16, 5301. 10.1038/s41467-025-60554-2. 40506454 PMC12162856

[116] Smith PM, Howitt MR, Panikov N, Michaud M, Gallini CA, Bohlooly-Y M, Glickman JN, and Garrett WS (2013). The microbial metabolites, short chain fatty acids, regulate colonic Treg cell homeostasis. Science 341, 569–573. 10.1126/science.1241165. 23828891 PMC3807819

[117] Hirshfield AN (1985). Comparison of Granulosa Cell Proliferation in Small Follicles of Hypophysectomized, Prepubertal, and Mature Rats1. Biol. Reprod 32, 979–987. 10.1095/biolreprod32.4.979. 4039955

[118] Hirshfield AN (1988). Size-frequency analysis of atresia in cycling rats. Biol. Reprod 38, 1181–1188. 10.1095/biolreprod38.5.1181. 3408785

[119] Gosden RG; Telfer (1987). Numbers of follicles and oocytes in mammalian ovaries and their allometric relationships. J. Zool 211, 169–175. 10.1111/j.1469-7998.1987.tb07460.x.

[120] Gosden RG; Telfer (1987). Scaling of follicular sizes in mammalian ovaries. J. Zool 211, 157–168. 10.1111/j.1469-7998.1987.tb07459.x.

[121] Brieño-Enríquez MA, Faykoo-Martinez M, Goben M, Grenier JK, McGrath A, Prado AM, Sinopoli J, Wagner K, Walsh PT, Lopa SH, (2023). Postnatal oogenesis leads to an exceptionally large ovarian reserve in naked mole-rats. Nat. Commun 14, 670. 10.1038/s41467-023-36284-8. 36810851 PMC9944903

[122] Arnold TW (2010). Uninformative Parameters and Model Selection Using Akaike’s Information Criterion. J. Wildl. Manag 74, 1175–1178. 10.1111/j.1937-2817.2010.tb01236.x.

[123] Staples TL (2023). Expansion and evolution of the R programming language. R. Soc. Open Sci 10, 221550. 10.1098/rsos.221550. 37063989 PMC10090872

[124] Callahan BJ, McMurdie PJ, Rosen MJ, Han AW, Johnson AJA, and Holmes SP (2016). DADA2: High-resolution sample inference from Illumina amplicon data. Nat. Methods 13, 581–583. 10.1038/nmeth.3869. 27214047 PMC4927377

[125] Quast C, Pruesse E, Yilmaz P, Gerken J, Schweer T, Yarza P, Peplies J, and Glockner FO (2013). The SILVA ribosomal RNA gene database project: improved data processing and web-based tools. Nucleic Acids Res. 41, D590–D596. 10.1093/nar/gks1219. 23193283 PMC3531112

[126] Katoh K, and Standley DM (2013). MAFFT Multiple Sequence Alignment software version 7: improvements in performance and usability. Mol. Biol. Evol 30, 772–780. 10.1093/molbev/mst010. 23329690 PMC3603318

[127] Price MN, Dehal PS, and Arkin AP (2010). FastTree 2 – Approximately Maximum-Likelihood Trees for Large Alignments. PLOS One 5, e9490. 10.1371/journal.pone.0009490. 20224823 PMC2835736

[128] McMurdie PJ, and Holmes S (2013). phyloseq: An R Package for Reproducible Interactive Analysis and Graphics of Microbiome Census Data. PLoS One 8, e61217. 10.1371/journal.pone.0061217. 23630581 PMC3632530

[129] Davis NM, Proctor DM, Holmes SP, Relman DA, and Callahan BJ (2018). Simple statistical identification and removal of contaminant sequences in marker-gene and metagenomics data. Microbiome 6, 226. 10.1186/s40168-018-0605-2. 30558668 PMC6298009

[130] Callahan BJ, Sankaran K, Fukuyama JA, McMurdie PJ, and Holmes SP (2016). Bioconductor Workflow for Microbiome Data Analysis: from raw reads to community analyses. F1000Res 5, 1492. 10.12688/f1000research.8986.2. 27508062 PMC4955027

[131] Bisanz JE, Upadhyay V, Turnbaugh JA, Ly K, and Turnbaugh PJ (2019). Meta-Analysis Reveals Reproducible Gut Microbiome Alterations in Response to a High-Fat Diet. Cell Host Microbe 26, 265–272.e4. 10.1016/j.chom.2019.06.013. 31324413 PMC6708278

[132] Weber N, Liou D, Dommer J, MacMenamin P, Quiñones M, Misner I, Oler AJ, Wan J, Kim L, Coakley McCarthy M, (2018). Nephele: a cloud platform for simplified, standardized and reproducible microbiome data analysis. Bioinformatics 34, 1411–1413. 10.1093/bioinformatics/btx617. 29028892 PMC5905584

[133] Bolger AM, Lohse M, and Usadel B (2014). Trimmomatic: a flexible trimmer for Illumina sequence data. Bioinformatics 30, 2114–2120. 10.1093/bioinformatics/btu170. 24695404 PMC4103590

[134] Langdon WB (2015). Performance of genetic programming optimised Bowtie2 on genome comparison and analytic testing (GCAT) benchmarks. BioData Min. 8, 1. 10.1186/s13040-014-0034-0. 25621011 PMC4304608

[135] Lu J, and Salzberg SL (2020). Ultrafast and accurate 16S rRNA microbial community analysis using Kraken 2. Microbiome 8, 124. 10.1186/s40168-020-00900-2. 32859275 PMC7455996

[136] Hyatt D, Chen G-L, LoCascio PF, Land ML, Larimer FW, and Hauser LJ (2010). Prodigal: prokaryotic gene recognition and translation initiation site identification. BMC Bioinformatics 11, 119. 10.1186/1471-2105-11-119. 20211023 PMC2848648

[137] Aoki KF, and Kanehisa M (2005). Using the KEGG Database Resource. Curr. Protoc. Bioinformatics 11, 1.12.1–1.12.54. 10.1002/0471250953.bi0112s11. 18428742

[138] Kanehisa M (2002). The KEGG Database. In ‘In Silico’ Simulation of Biological Processes, Bock G and Goode JA, eds. (John Wiley & Sons), pp. 91–103. 10.1002/0470857897.ch8.

[139] Munyoki SK, Goff JP, Kolobaric A, Long A, Mullett SJ, Burns JK, Jenkins AK, DePoy L, Wendell SG, McClung CA, (2023). Intestinal microbial circadian rhythms drive sex differences in host immunity and metabolism. iScience 26, 107999. 10.1016/j.isci.2023.107999. 37841582 PMC10568425

[140] Han J, Lin K, Sequeira C, and Borchers CH (2015). An isotopelabeled chemical derivatization method for the quantitation of short-chain fatty acids in human feces by liquid chromatography–tandem mass spectrometry. Anal. Chim. Acta 854, 86–94. 10.1016/j.aca.2014.11.015. 25479871

[141] Babraham Bioinformatics. FastQC A Quality Control tool for High Throughput Sequence Data. https://www.bioinformatics.babraham.ac.uk/projects/fastqc/.

[142] Ewels P, Magnusson M, Lundin S, and Kaller M (2016). MultiQC: summarize analysis results for multiple tools and samples in a single report. Bioinformatics 32, 3047–3048. 10.1093/bioinformatics/btw354. 27312411 PMC5039924

[143] Bray NL, Pimentel H, Melsted P, and Pachter L (2016). Near-optimal probabilistic RNA-seq quantification. Nat. Biotechnol 34, 525–527. 10.1038/nbt.3519. 27043002

[144] Robinson MD, McCarthy DJ, and Smyth GK (2010). edgeR: a Bioconductor package for differential expression analysis of digital gene expression data. Bioinformatics 26, 139–140. 10.1093/bioinformatics/btp616. 19910308 PMC2796818

[145] Ritchie ME, Phipson B, Wu D, Hu Y, Law CW, Shi W, and Smyth GK (2015). limma powers differential expression analyses for RNA-sequencing and microarray studies. Nucleic Acids Res. 43, e47. 10.1093/nar/gkv007. 25605792 PMC4402510

[146] Murrell P (2009). R Graphics. WIREs Comp. Stat 1, 216–220. 10.1002/wics.22.

[147] Galili T, O’Callaghan A, Sidi J, and Sievert C (2018). heatmaply: an R package for creating interactive cluster heatmaps for online publishing. Bioinformatics 34, 1600–1602. 10.1093/bioinformatics/btx657. 29069305 PMC5925766

[148] Xu S, Hu E, Cai Y, Xie Z, Luo X, Zhan L, Tang W, Wang Q, Liu B, Wang R, (2024). Using clusterProfiler to characterize multiomics data. Nat. Protoc 19, 3292–3320. 10.1038/s41596-024-01020-z. 39019974

[149] Yu G, Wang L-G, Han Y, and He Q-Y (2012). clusterProfiler: an R package for comparing biological themes among gene clusters. Omics 16, 284–287. 10.1089/omi.2011.0118. 22455463 PMC3339379

[150] Paxinos G, and Franklin KBJ (2019). Paxinos and Franklin’s the Mouse Brain in Stereotaxic Coordinates (Academic Press).

