# Supplementary material for: The microbiota extends the reproductive lifespan of mice by safeguarding the ovarian reserve
Source: Cell Host Microbe. Author manuscript; Available in PMC 2026 Jul 16. (PMC13374525; doi:10.1016/j.chom.2025.09.006)
Supplement: MMC1 [file NIHMS2110120-supplement-MMC1.pdf]

Cell Host & Microbe, Volume *33*

Supplemental information

The microbiota extends the reproductive lifespan

# of mice by safeguarding the ovarian reserve

Sarah K. Munyoki, Julie P. Goff, Amanda Reshke, Erin Wilderoter, Nyasha Mafarachisi, Antonija Kolobaric, Yi Sheng, Steven J. Mullett, Gabrielle E. King, Jacob D. DeSchepper, Richard J. Bookser, Carlos A. Castro, Stacy L. Gelhaus, Mayara Grizotte-Lake, Kathleen E. Morrison, Anthony J. Zeleznik, Timothy W. Hand, Miguel A. Brieño-Enriquez, and Eldin Jašarević

Supplemental information

The microbiota extends the reproductive lifespan of mice by safeguarding the ovarian reserve

Sarah K. Munyoki, Julie P. Goff, Amanda Reshke, Erin Wilderoter, Nyasha Mafarachisi, Antonija Kolobaric, Yi Sheng, Steven J. Mullett, Gabrielle E. King, Jacob D. DeSchepper, Richard J. Bookser, Carlos A. Castro, Stacy L. Gelhaus, Mayara Grizotte-Lake, Kathleen E. Morrison, Anthony J. Zeleznik, Timothy W. Hand, Miguel A. Brieño-Enriquez, and Eldin Jašarević

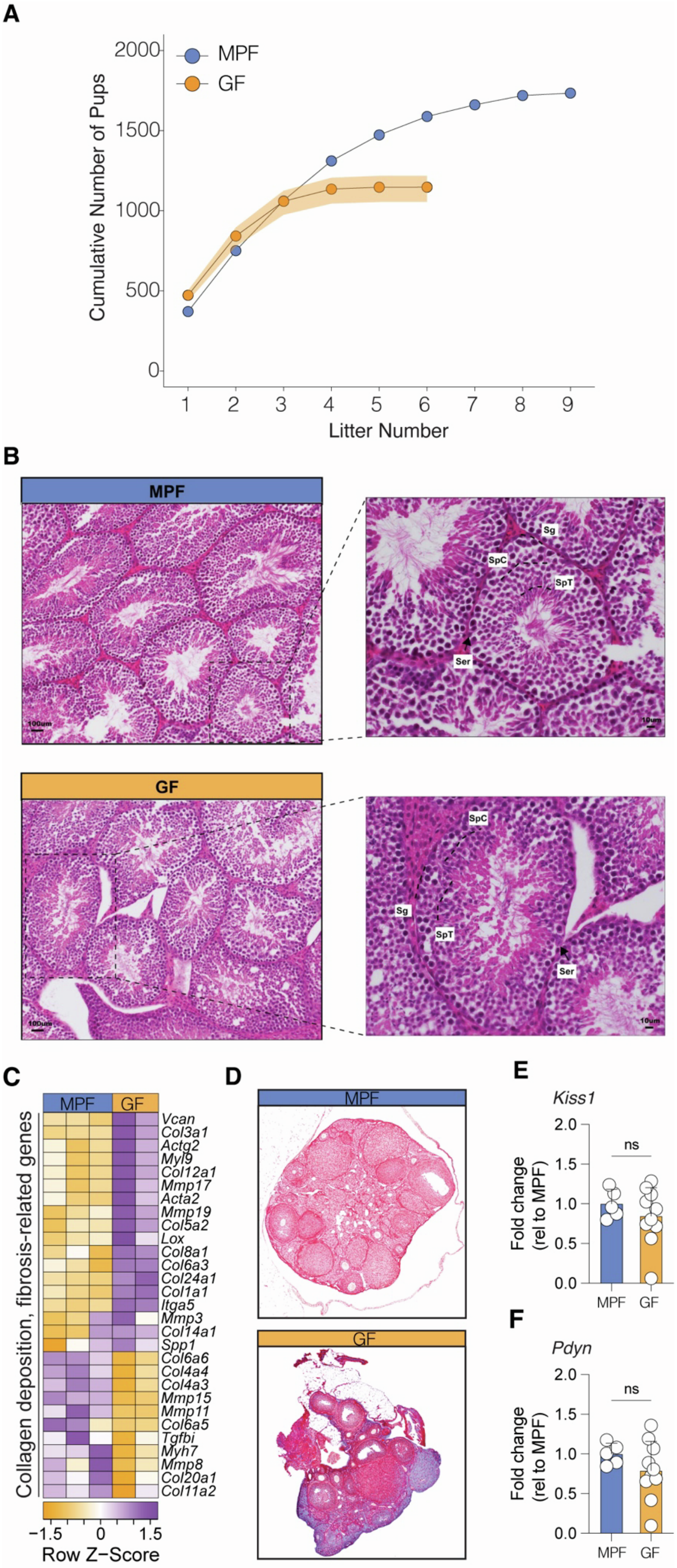

Supplemental Figure 1. The microbiota extends reproductive lifespan and influences ovarian morphology and gene expression (Related to Figures 1 and 2)

(A) Undersampling analysis (1000 permutations) of the cumulative number of pups produced by MPF (blue) and GF (orange) female mice over successive litters. MPF mice show continued reproductive output beyond the 5th litter, while GF mice reproduction plateaus after the 5th litter. Data shown as mean ± 95% confidence intervals. MPF (n = 58), GF (n = 89), totaling 493 litters.

(B) Representative ovarian histology from 10-week-old MPF (top) and GF (bottom) female mice. H&E staining, scale bars: 100 µm. The inset shows a higher magnification of the detailed cellular organization of seminiferous tubules, featuring spermatogonia (Sg), spermatocytes (SpC), spermatids (SpT), and Sertoli cells (Ser). Scale bars: 10 µm. Both MPF and GF testes exhibit active spermatogenesis, characterized by comparable cellular architecture and organization.

(C) Heatmap of collagen deposition and fibrosis-related gene expression in MPF and GF ovaries at 10 weeks (FDR < 0.25). Data displayed as row Z-scores. GF ovaries show higher expression of several collagen and ECM-related genes compared to MPF. MPF (n = 3), GF (n = 2).

(D) Representative Masson's trichrome staining of ovarian sections from MPF (top) and GF (bottom) mice. Blue staining indicates collagen deposition. GF ovary displays increased collagen deposition and fibrotic changes compared to MPF. Scale bars: 100 µm. MPF (n = 4), GF (n = 5).

(E) Fold change in *Kiss1* mRNA transcript levels in the arcuate nucleus of the hypothalamus of MPF and GF mice at P70. Unpaired t-test (ns). MPF (n = 5), GF (n = 10).

(F) Fold change in *Pdyn* mRNA transcript levels in the arcuate nucleus of the hypothalamus of MPF and GF mice at P70. Unpaired t-test (ns). MPF (n = 5), GF (n = 10).

Data shown as mean ± SD unless otherwise noted. MPF, murine pathogen-free; GF, germ-free; ECM, extracellular matrix; P70, postnatal day 70. See also Figs. 1 and 2.

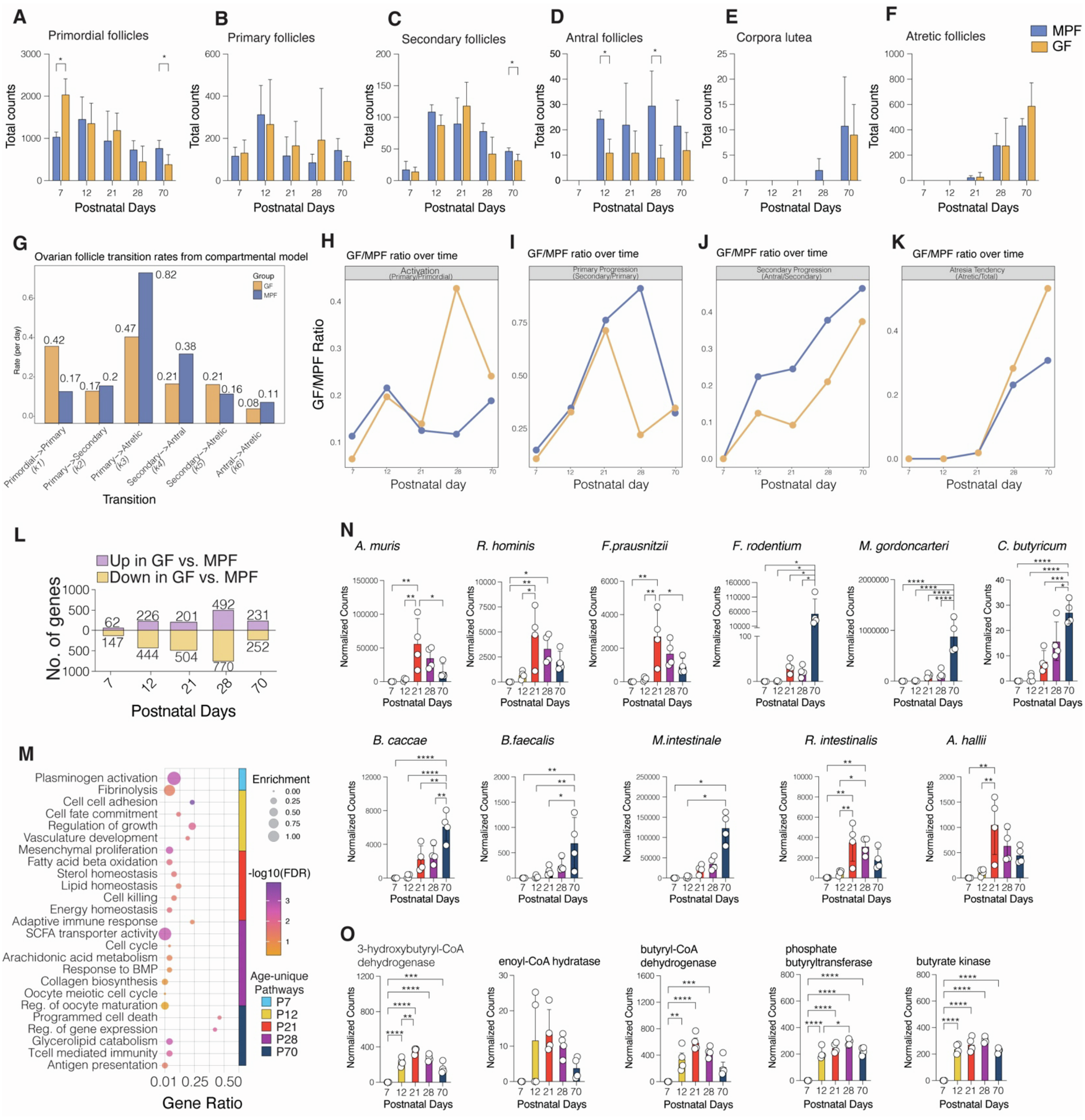

Supplemental Figure 2. Ovarian follicle dynamics, transcriptomic changes, and microbiota development during postnatal maturation (Related to Figure 3)

(A-F) Ovarian follicle counts in MPF and GF mice across postnatal development. **(A)** Primordial follicle counts. Unpaired t-test per time point (*p < 0.05). **(B)** Primary follicle counts. Unpaired t-test per time point (ns). **(C)** Secondary follicle counts. Unpaired t-test per time point (ns). **(D)** Antral follicle counts. Unpaired t-test per time point **(ns)**. (E) Corpora lutea counts. Unpaired t-test per time point (ns). **(F)** Atretic follicle counts. Unpaired t-test per time point (*$p < 0.05$). Sample sizes for each timepoint: P7 (MPF n = 4, GF n = 4), P12 (MPF n = 4, GF n = 4), P21 (MPF n = 4, GF n = 4), P28 (MPF n = 4, GF n = 4), P70 (MPF n = 4, GF n = 5).

(G) Ovarian follicle transition rates derived from a compartmental model comparing MPF and GF mice. The model quantifies six key transition rates: *k1* (Primordial to Primary, rate of primordial follicle activation), *k2* (Primary to Secondary), *k3* (Primary to Atretic), *k4* (Secondary to Antral), *k5* (Secondary to Atretic), and k6 (Antral to Atretic). Bar heights represent calculated

daily rates (per day) for each transition. GF mice exhibit higher primordial follicle activation rates (*k1*) and primary follicle atresia (*k3*) but altered progression through later stages.

(H-K) Follicle transition ratios over postnatal development. **(H)** Activation ratio (Primary/Primordial). **(I)** Primary progression ratio (Secondary/Primary). **(J)** Secondary progression ratio (Antral/Secondary). **(K)** Atresia tendency (Atretic/Total follicles). Data derived from histological ovarian count data. Sample sizes: P7 (MPF n = 4, GF n = 3), P12 (MPF n = 4, GF n = 4), P21 (MPF n = 4, GF n = 4), P28 (MPF n = 4, GF n = 4), P70 (MPF n = 4, GF n = 5).

(L) Quantification of differentially expressed genes (FDR < 0.25) in ovaries of GF compared to MPF mice across postnatal development. Purple: upregulated in GF; yellow: downregulated. Sample sizes: P7 (MPF n = 3, GF n = 3), P12 (MPF n = 3, GF n = 4), P21 (MPF n = 2, GF n = 2), P28 (MPF n = 3, GF n = 3), P70 (MPF n = 3, GF n = 5).

(M) Functional enrichment analysis of Gene Ontology Biological Process terms of age-specific downregulated genes in GF ovaries. Bubble size represents enrichment score; color intensity indicates statistical significance (-log10FDR). The sidebar indicates specific developmental timepoints where each pathway is uniquely dysregulated.

(N) Normalized abundance of SCFA-producing bacterial species across postnatal development (P7, P12, P21, P28, P70) in MPF mice: *A. muris, R. hominis, F. prausnitzii, F. rodentium, M. gordoncarteri, C. butyricum, B. caccae, B. faecalis, M. intestinale, R. intestinalis,* and *A. hallii.* One-way ANOVA with Tukey's test (*p < 0.05, ***p* < 0.01, ****p* < 0.001, *****p* < 0.0001).

(O) Normalized abundance of genes involved in butyrate production across postnatal development (P7, P12, P21, P28, P70) in MPF mice: 3-hydroxybutyryl-CoA dehydrogenase, enoyl-CoA hydratase, butyryl-CoA dehydrogenase, phosphate butyryltransferase, and butyrate kinase. One-way ANOVA with Tukey's test (*p < 0.05, ***p* < 0.01, ****p* < 0.001, *****p* < 0.0001). Sample size for panels N-O: P7 (n = 4), P12 (n = 4), P21 (n = 4), P28 (n = 4), P70 (n = 4).

Data shown as mean ± SD unless specified. MPF, murine pathogen-free; GF, germ-free; P7-P70, postnatal days; SCFA, short-chain fatty acid; FDR, false discovery rate. See also Fig. 3.

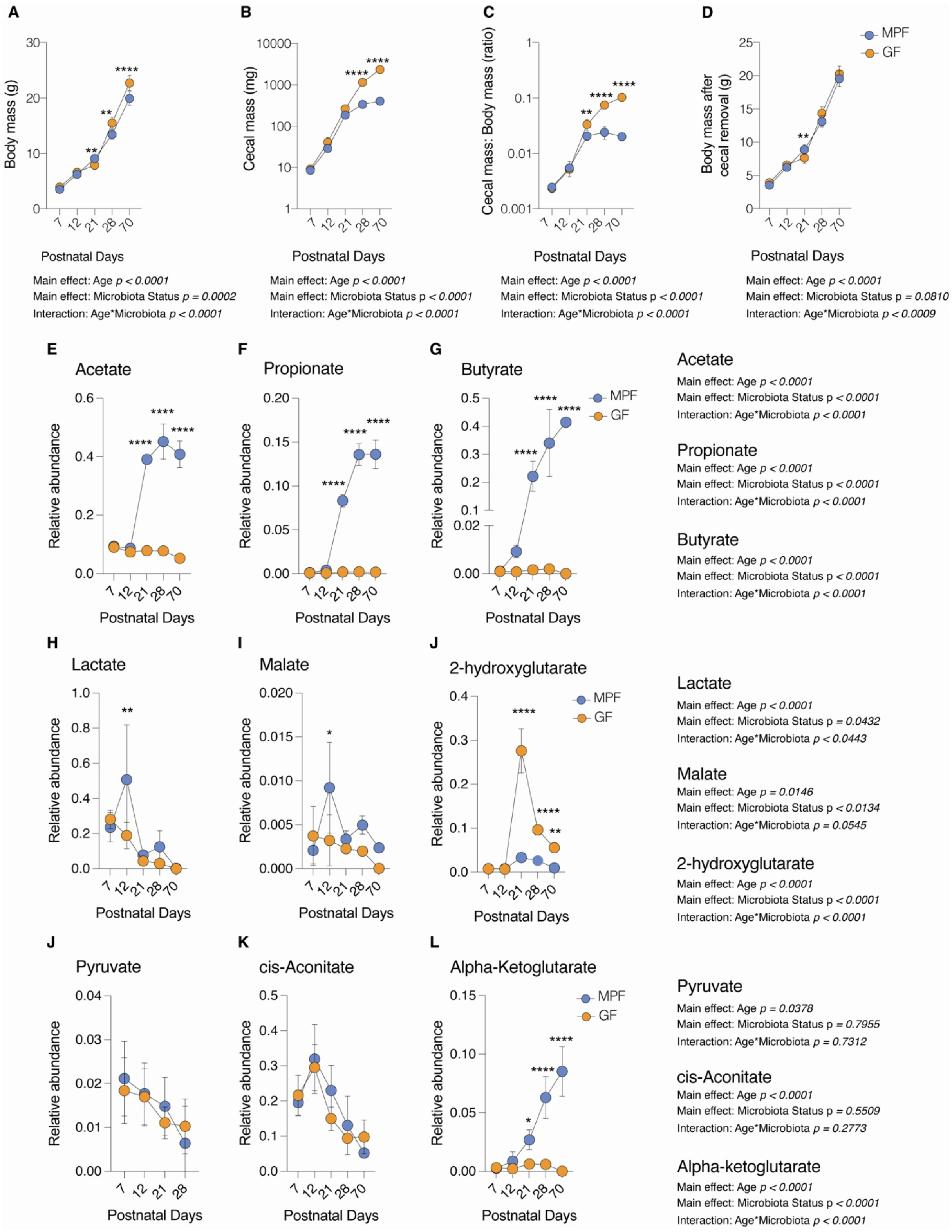

Supplemental Figure 3. Gut microbiota influences metabolic parameters across postnatal development (Related to Figure 3)

(A) Body mass of MPF and GF female mice across postnatal development. Two-way ANOVA with Šidák's test. Main effect: Age $p < 0.0001$; Main effect: Microbiota Status $p = 0.0002$; Interaction: Age*Microbiota $p < 0.0001$.

(B) Cecal mass of MPF and GF female mice across postnatal development. Two-way ANOVA with Šidák's test. Main effect: Age $p < 0.0001$; Main effect: Microbiota Status $p < 0.0001$; Interaction: Age*Microbiota $p < 0.0001$.

(C) Cecal mass to body mass ratio of MPF and GF female mice across postnatal development. Two-way ANOVA with Šidák's test. Main effect: Age $p < 0.0001$; Main effect: Microbiota Status $p < 0.0001$; Interaction: Age*Microbiota $p < 0.0001$.

(D) Body mass after cecal removal of MPF and GF female mice across postnatal development. Two-way ANOVA with Šidák's test. Main effect: Age $p < 0.0001$; Main effect: Microbiota Status $p = 0.0810$; Interaction: Age*Microbiota p < 0.0009.

(E-G) Short-chain fatty acid levels in cecal contents across postnatal development. **(E)** Acetate levels. **(F)** Propionate levels. **(G)** Butyrate levels. Two-way ANOVA with Šidák's test. All SCFAs exhibit significant main effects of age and microbiota status, with significant interactions (all *p value*s < 0.0001).

(H-M) Additional metabolite levels in cecal contents across postnatal development. **(H)** Lactate levels. Main effect: Age p < 0.0001; Main effect: Microbiota Status $p = 0.0432$; Interaction: Age × Microbiota, $p = 0.0443$. **(I)** Malate levels. Main effect: Age $p = 0.0146$; Main effect: Microbiota Status $p < 0.0134$; Interaction: Age × Microbiota, $p = 0.0545$**.** **(J)** 2-hydroxyglutarate levels. Main effects: Age, $p < 0.0001$; Microbiota Status, $p < 0.0001$; Interaction: Age × Microbiota, $p < 0.0001$. **(K)** Pyruvate levels. Main effects: Age, $p = 0.0376$; Main effect: Microbiota Status, $p = 0.7955$; Interaction: Age × Microbiota, $p = 0.7312$. **(L)** cis-Aconitate levels. Main effect: Age p < 0.0001; Main effect: Microbiota Status $p = 0.5509$; Interaction: Age × Microbiota, p = 0.2773. **(M)** Alpha-ketoglutarate levels. Main effect: Age, $p < 0.0001$; Microbiota Status, p < 0.0001; Interaction: Age × Microbiota, p < 0.0001. Two-way ANOVA with Šidák's test for all panels.

Sample sizes: For panels A-D: P7 (MPF n = 4, GF n = 8), P12 (MPF n = 6, GF n = 4), P21 (MPF n = 13, GF n = 2), P28 (MPF n = 6, GF n = 4), P70 (MPF n = 6, GF n = 4). For panels E-M: P7 (MPF n = 4, GF n = 5), P12 (MPF n = 3, GF n = 3), P21 (MPF n = 3, GF n = 3), P28 (MPF n = 3, GF n = 3), P70 (MPF n = 3, GF n = 3). Data shown as mean ± SD. *p < 0.05, **p < 0.01, ***p < 0.001, ****p < 0.0001. MPF, murine pathogen-free; GF, germ-free; SCFA, short-chain fatty acid. See also Fig. 3.

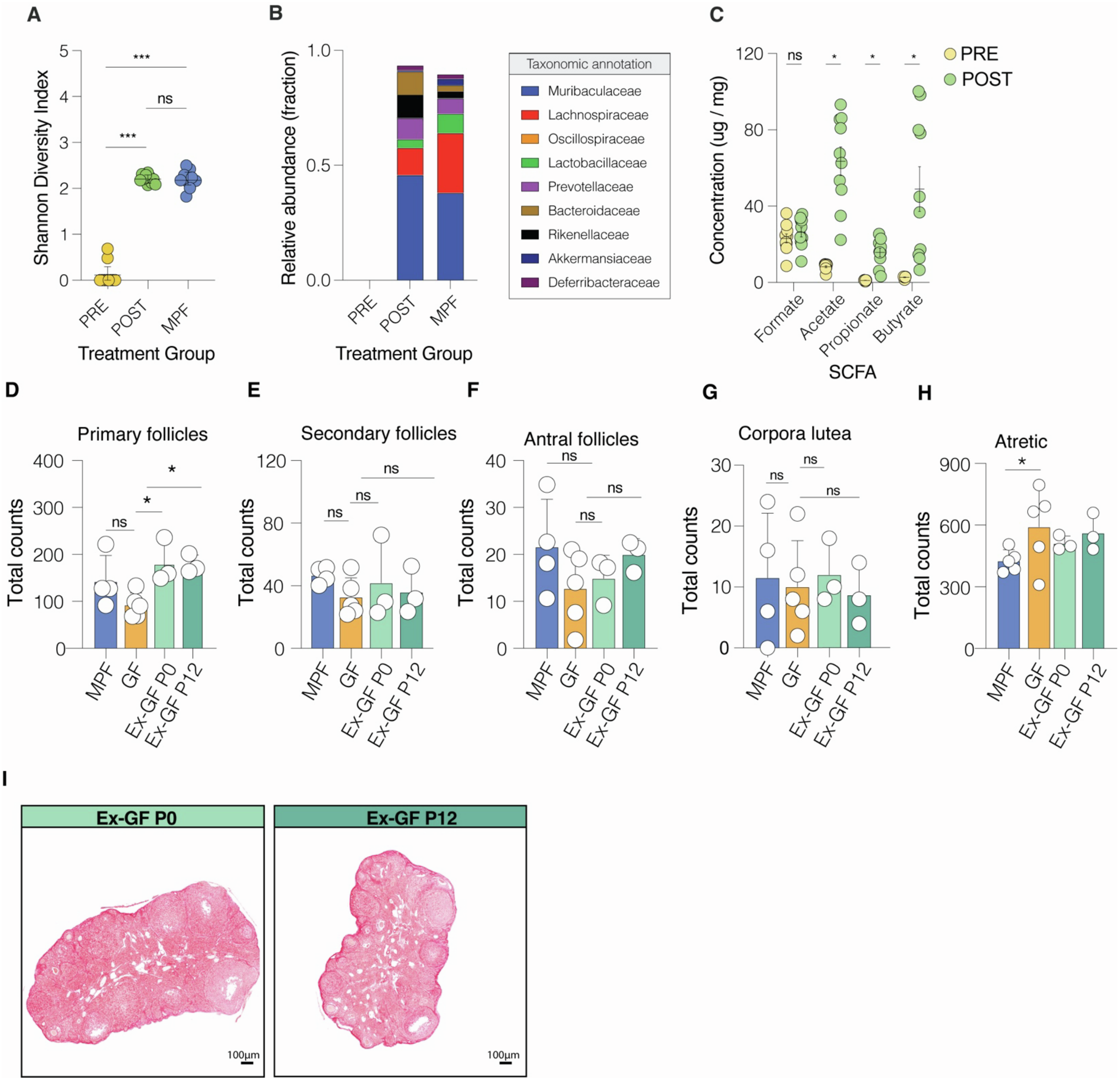

Supplemental Figure 4. Early-life microbiota colonization mediates ovarian follicle dynamics (Related to Figure 4)

(A) Shannon Diversity Index of cecal microbiota in PRE-colonization, POST-colonization, and MPF female mice. One-way ANOVA with Tukey's test (***p < 0.001, ns, not significant).

(B) Relative abundance (fraction) of major bacterial taxa in cecal microbiota of PRE-colonization, POST-colonization, and MPF female mice.

(C) Concentrations of SCFAs in cecal contents of PRE-colonization and POST-colonization female mice. Unpaired t-test (*p < 0.05). Sample size for panels A-C: 10 colonized GF dams, 8 MPF dams. PRE, before colonization; POST, 7 days post-colonization.

(D) Primary follicle counts in MPF, Ex-GF P0-colonized, Ex-GF P12-colonized, and GF female mice at P70. One-way ANOVA with Fisher's LSD test (*p < 0.05).

(E) Secondary follicle counts in MPF, Ex-GF P0-colonized, Ex-GF P12-colonized, and GF female mice at P70. One-way ANOVA with Fisher's LSD test (ns).

(F) Antral follicle counts in MPF, Ex-GF P0-colonized, Ex-GF P12-colonized, and GF female mice at P70. One-way ANOVA with Fisher's LSD test (ns).

(G) Corpora lutea counts in MPF, Ex-GF P0-colonized, Ex-GF P12-colonized, and GF female mice at P70. One-way ANOVA with Fisher's LSD test (ns).

(H) Atretic follicle counts in MPF, Ex-GF P0-colonized, Ex-GF P12-colonized, and GF female mice at P70. One-way ANOVA with Fisher's LSD test (*$p < 0.05$).

(I) Representative Masson's Trichrome-stained ovarian sections from Ex-GF P0-colonized and Ex-GF P12-colonized female mice at P70. Scale bars: 100 μm.

Sample sizes for panels D-I: MPF (n = 4), GF (n = 5), Ex-GF P0 (n = 3), Ex-GF P12 (n = 3). Data shown as mean ± SD. MPF, murine pathogen-free; GF, germ-free; Ex-GF, formerly germ-free; P0, colonized at birth; P12, colonized at postnatal day 12; P70, postnatal day 70; SCFA, short-chain fatty acids. See also Fig. 4.

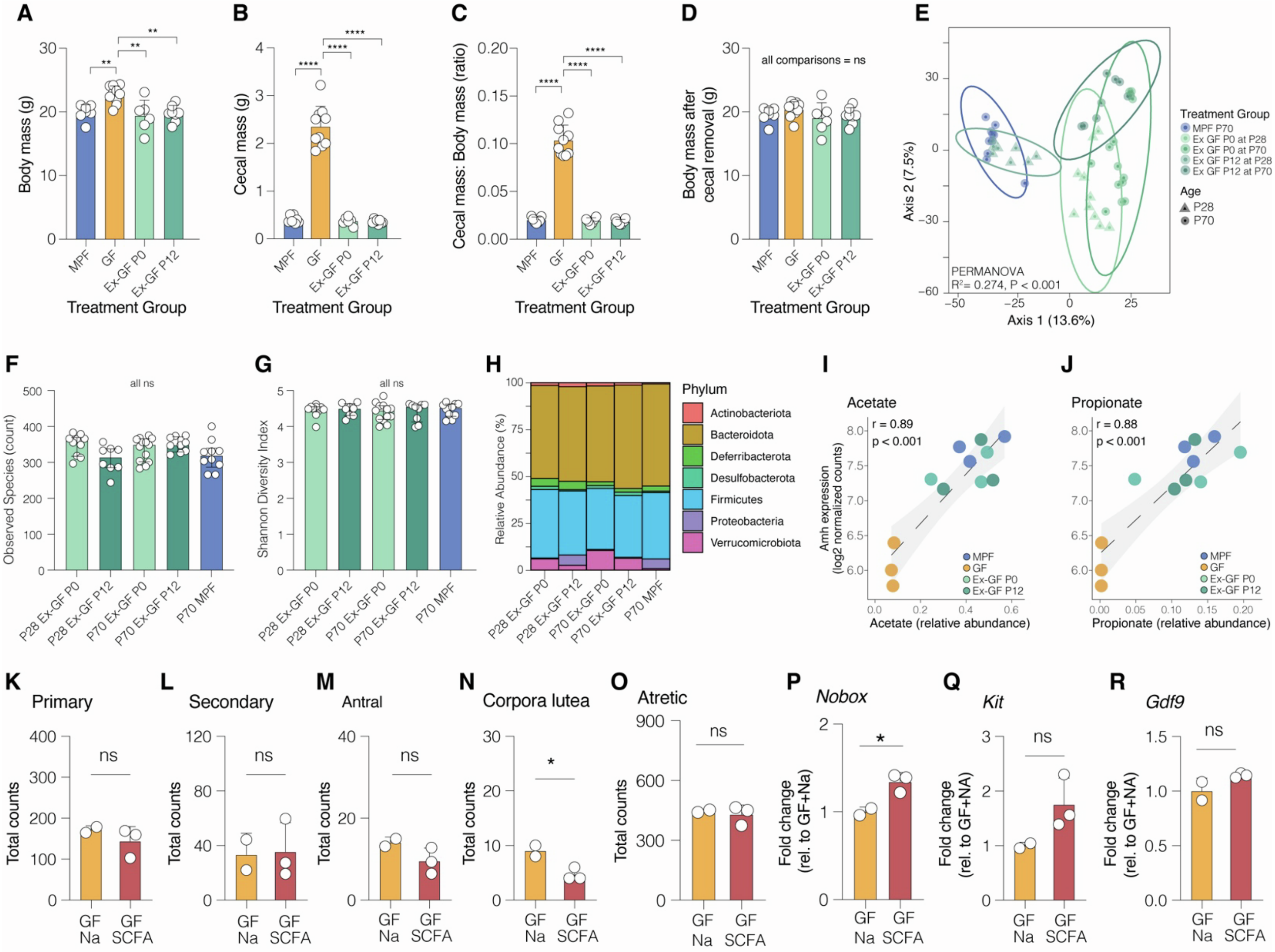

Supplemental Figure 5. Early-life microbiota colonization normalizes cecal microbial composition and metabolic parameters, and additional characterization of SCFA supplementation effects on ovarian function (Related to Figure 4)

Early-life colonization effects on host physiology and microbiota (A-J):

(A) Body mass in MPF, GF, Ex-GF P0-colonized, and Ex-GF P12-colonized female mice at P70. One-way ANOVA with Tukey's test (**$p < 0.01$).

(B) Cecal mass in MPF, GF, Ex-GF P0-colonized, and Ex-GF P12-colonized female mice at P70. One-way ANOVA with Tukey's test (****$p < 0.0001$).

(C) Cecal mass to body mass ratio in MPF, GF, Ex-GF P0-colonized, and Ex-GF P12-colonized female mice at P70. One-way ANOVA with Tukey's test (****$p < 0.0001$).

(D) Body mass after cecal removal in MPF, GF, Ex-GF P0-colonized, and Ex-GF P12-colonized female mice at P70. One-way ANOVA (all comparisons ns). Sample sizes for panels A-D: MPF (n = 6), GF (n = 10), Ex-GF P0 (n = 6), Ex-GF P12 (n = 8).

(E) Principal coordinates analysis of cecal microbiota composition (16S rRNA) showing clustering by colonization treatment and age. PERMANOVA, $R^2 = 0.274$, **$p < 0.001$.

(F) Observed species count in P28 and P70 Ex-GF P0, Ex-GF P12, and MPF P70 mice. Kruskal-Wallis test (all ns).

(G) Shannon Diversity Index in P28 and P70 Ex-GF P0, Ex-GF P12, and MPF P70 mice. Kruskal-Wallis test (all ns).

(H) Relative abundance of major bacterial phyla in P28 and P70 Ex-GF P0, Ex-GF P12, and MPF P70 mice.

Sample sizes for panels E-H: MPF P70 (n = 6), Ex-GF P0 at P28 (n = 6), Ex-GF P0 at P70 (n = 6), Ex-GF P12 at P28 (n = 8), Ex-GF P12 at P70 (n = 8).

(I) Correlation between ovarian *Amh* expression and cecal acetate abundance. Pearson correlation, r = 0.89, $p < 0.001$.

(J) Correlation between ovarian *Amh* expression and cecal propionate abundance. Pearson correlation, r = 0.88, $p < 0.001$. Sample sizes for panels I-J: MPF (n = 3), GF (n = 3), Ex-GF P0 (n = 3), Ex-GF P12 (n = 3).

SCFA supplementation effects (K-R):

(K) Primary follicle counts in GF+Vehicle and GF+SCFA female mice at P70. Unpaired t-test (ns).

(L) Secondary follicle counts in GF+Vehicle and GF+SCFA female mice at P70. Unpaired t-test (ns).

(M) Antral follicle counts in GF+Vehicle and GF+SCFA female mice at P70. Unpaired t-test (ns).

(N) Corpora lutea counts in GF+Vehicle and GF+SCFA female mice at P70. Unpaired t-test (*$p < 0.05$).

(O) Atretic follicle counts in GF+Vehicle and GF+SCFA female mice at P70. Unpaired t-test (ns).

(P) qPCR of ovarian tissue *Nobox* mRNA transcript levels. Unpaired t-test (*$p < 0.05$).

(Q) qPCR of ovarian tissue *Kit* mRNA transcript levels. Unpaired t-test (ns).

(R) qPCR of ovarian tissue *Gdf9* mRNA transcript levels. Unpaired t-test (ns). Sample sizes for panels K-R: GF+Vehicle (n = 2), GF+SCFA (n = 3).

Data shown as mean ± SD. *$p < 0.05$, **$p < 0.01$, ***$p < 0.001$, ****$p < 0.0001$, ns, not significant. MPF, murine pathogen-free; GF, germ-free; Ex-GF, formerly germ-free; P0, colonized at birth; P12, colonized at postnatal day 12; P28, postnatal day 28; P70, postnatal day 70; SCFA, short-chain fatty acids; Vehicle, pH- and sodium-matched control water. See also Fig. 4.

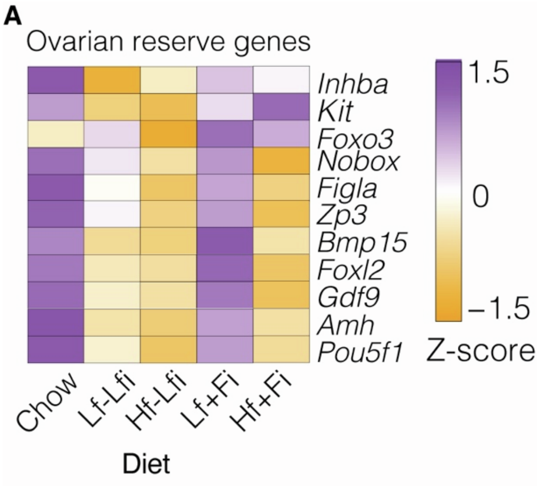

Supplemental Figure 6. Expression of genes involved in the maintenance of the ovarian reserve across dietary groups (Related to Figure 5)

(A) Heatmap showing expression of key ovarian reserve genes across different dietary groups. Fiber supplementation maintained expression at Chow-like levels in Lf+Fi mice, despite a reduction under Lf-Lfi conditions. A high-fat diet (Hf-Lfi) downregulated these genes, and fiber supplementation did not restore their expression, except for Kit and Inhba. Values represent Z-scores of normalized expression from whole-tissue ovary RNA-seq. Sample sizes: Chow (n = 5), Lf-Lfi (n = 3), Hf-Lfi (n = 3), Lf+Fi (n = 3), Hf+Fi (n = 3).

Data shown as Z-scores. Chow, standard chow diet; Lf-Lfi, low-fat diet with low fiber; Hf-Lfi, high-fat diet with low fiber; Lf+Fi, low-fat diet with fiber supplementation; Hf+Fi, high-fat diet with fiber supplementation. See also Fig. 5.

Supplemental Table 1. Statistical modeling of primordial follicle loss and atretic follicle accumulation in MPF and GF mice, Related to Figure 3.

Group	Follicle Type	Model Type	R^2	AIC	Model Equation
MPF	Primordial	Exponential	0.446	73.8	y = 1395.1 × e^(-0.121x)
MPF	Primordial	Power Function	0.338	75.7	y = 99132 × x^(-2.677) - 97893
MPF	Primordial	Linear	0.472	72.6	y = 1362.9 - 126.2x
GF	Primordial	Exponential	0.946	69.8	y = 3054.5 × e^(-0.394x)
GF	Primordial	Power Function	0.957	70.7	y = -1609.5 × x^(0.449) + 3633.6
GF	Primordial	Linear	0.942	70.2	y = 2346.2 - 421.3x
MPF	Atretic	Exponential	0.921	59.3	y = 6.94 × e^(-0.836x)
MPF	Atretic	Power Function	0.64	68.9	y = -26344.5 × x^(-9.491) + 26252.0
MPF	Atretic	Linear	0.83	63.1	y = -196.4 + 114.3x
GF	Atretic	Exponential	0.974	56.2	y = 4.02 × e^(-1.001x)
GF	Atretic	Power Function	0.597	72	y = -35470.1 × x^(-8.787) + 35349.7
GF	Atretic	Linear	0.799	66.5	y = -256.7 + 144.8x

Table S2. Curated gene sets for ovarian follicle regulatory pathways, related to Figure 3.

Gene	Pathway	Reference
Pten	Activation	PMID: 18239123
Foxo3	Activation	PMID: 12855809
Foxo1	Activation	PMID: 11875118
Tsc1	Activation	PMID: 19843540
Tsc2	Activation	PMID: 19843540
Stk11	Activation	PMID: 26745759
Rps6kb1	Activation	PMID: 14982927
Rps6	Activation	PMID: 14982927
Akt	Activation	PMID: 24082083
Pdk1	Activation	PMID: 19423553
Pik3ca	Activation	PMID: 19447101
Pik3r1	Activation	PMID: 19447101
Kitl	Activation	PMID: 10465300
Kit	Activation	PMID: 10465300
Amh	Activation	PMID: 10579345
Amhr2	Activation	PMID: 10579345
Bdnf	Activation	PMID: 15013804
Ntrk2	Activation	PMID: 15013804
Sohlh1	Activation	PMID: 16690745
Sohlh2	Activation	PMID: 16690745
Nobox	Activation	PMID: 15326356
Lhx8	Activation	PMID: 16690745
Figla	Activation	PMID: 11023867
Cdkn1b	Activation	PMID: 17565040
Cdkn1a	Activation	PMID: 12609976
Lin28a	Activation	PMID: 24715688
Lin28b	Activation	PMID: 24715688
Nppc	Activation	PMID: 20947764
Npr2	Activation	PMID: 20947764
Smad3	Activation	PMID: 11906909
Zp3	Activation	PMID: 8643592
Gdf9	Activation	PMID: 8849725
Bmp15	Activation	PMID: 11376106
Jag1	Activation	PMID: 24552588
Notch2	Activation	PMID: 24552588
Fshr	Progression	PMID: 9020850
Cyp19a1	Progression	PMID: 11431142
Lhcgr	Progression	PMID: 11145749
Cyp17a1	Progression	PMID: 15269096
Cyp11a1	Progression	PMID: 15269096
Star	Progression	PMID: 9326645
Hsd3b1	Progression	PMID: 15583024
Hsd17b1	Progression	PMID: 15583024
Nr5a1	Progression	PMID: 15118069
Nr5a2	Progression	PMID: 18628394
Inha	Progression	PMID: 1448148
Inhba	Progression	PMID: 1448148
Inhbb	Progression	PMID: 1448148
Fst	Progression	PMID: 7885475
Bmp6	Progression	PMID: 11416163
Bmpr1a	Progression	PMID: 11416163
Bmpr1b	Progression	PMID: 11416163
Bmpr2	Progression	PMID: 11416163
Smad1	Progression	PMID: 17967875
Smad5	Progression	PMID: 17967875
Smad4	Progression	PMID: 17967875
Tgfbr1	Progression	PMID: 22028666

Igf1	Progression	PMID: 9415397
Igf1r	Progression	PMID: 9415397
Igfbp2	Progression	PMID: 9415397
Igfbp4	Progression	PMID: 9415397
Egfr	Progression	PMID: 14726596
Ereg	Progression	PMID: 14726596
Areg	Progression	PMID: 14726596
Btc	Progression	PMID: 14726596
Foxl2	Progression	PMID: 14736745
Esr1	Progression	PMID: 8248223
Esr2	Progression	PMID: 8248223
Ar	Progression	PMID: 15277682
Pgr	Progression	PMID: 7557380
Wnt4	Progression	PMID: 9989404
Wnt2	Progression	PMID: 9989404
Ctnnb1	Progression	PMID: 16256976
Lef1	Progression	PMID: 16256976
Ccnd2	Progression	PMID: 8945475
Cdk4	Progression	PMID: 8945475
Pcna	Progression	PMID: 7492681
Ptgs2	Progression	PMID: 9346237
Ptger2	Progression	PMID: 9346237
Adamts1	Progression	PMID: 20592310
Tnfaip6	Progression	PMID: 12668637
Has2	Progression	PMID: 12668637
Gja1	Progression	PMID: 12668637
Gjc1	Progression	PMID: 12668637
Gja4	Progression	PMID: 12668637
Bax	Atresia	PMID: 7569956
Bcl2	Atresia	PMID: 7569956
Bcl2l1	Atresia	PMID: 7828536
Bcl2l11	Atresia	PMID: 7828536
Bak1	Atresia	PMID: 7828536
Mcl1	Atresia	PMID: 7828536
Bok	Atresia	PMID: 7828536
Bad	Atresia	PMID: 7828536
Bid	Atresia	PMID: 7828536
Bbc3	Atresia	PMID: 7828536
Pmaip1	Atresia	PMID: 7828536
Casp3	Atresia	PMID: 11356696
Casp6	Atresia	PMID: 11356696
Casp7	Atresia	PMID: 11356696
Casp8	Atresia	PMID: 11356696
Casp9	Atresia	PMID: 11356696
Trp53	Atresia	PMID: 25112877
Fas	Atresia	PMID: 9110309
Fasl	Atresia	PMID: 9110309
Tnfsf10	Atresia	PMID: 15574753
Tnfrsf10b	Atresia	PMID: 15574753
Xiap	Atresia	PMID: 19473982
Birc5	Atresia	PMID: 19473982
Diablo	Atresia	PMID: 19473982
Map3k5	Atresia	PMID: 15705959
Mapk8	Atresia	PMID: 15705959
Mapk9	Atresia	PMID: 15705959
Mapk14	Atresia	PMID: 15705959
Hif1a	Atresia	PMID: 24855100
Atg5	Atresia	PMID: 21464117
Atg7	Atresia	PMID: 21464117
Becn1	Atresia	PMID: 21464117
Map1lc3a	Atresia	PMID: 21464117
Map1lc3b	Atresia	PMID: 21464117

Sqstm1	Atresia	PMID: 21464117
Ulk1	Atresia	PMID: 21464117
Ripk1	Atresia	PMID: 30665407
Ripk3	Atresia	PMID: 30665407
Mlkl	Atresia	PMID: 30665407
Tnf	Atresia	PMID: 8985665
Tnfrsf1a	Atresia	PMID: 8985665
Aifm1	Atresia	PMID: 26063804
Cradd	Atresia	PMID: 26063804
Dapk1	Atresia	PMID: 26063804
Gadd45a	Atresia	PMID: 12960025
Gadd45b	Atresia	PMID: 12960025
